# Polypore fungi as a flagship group to indicate changes in biodiversity – a test case from Estonia

**DOI:** 10.1186/s43008-020-00050-y

**Published:** 2021-01-18

**Authors:** Kadri Runnel, Otto Miettinen, Asko Lõhmus

**Affiliations:** 1 Department of Zoology, Institute of Ecology and Earth Sciences, University of Tartu, Vanemuise 46, 51005 Tartu, Estonia; 2 Botanical Unit (Mycology), Finnish Museum of Natural History, University of Helsinki, Unioninkatu 44, 00170 Helsinki, Finland

**Keywords:** Assemblage composition, Cryptic species, Functional groups, Species pool, Substrate ecology, Wood-inhabiting fungi

## Abstract

Polyporous fungi, a morphologically delineated group of *Agaricomycetes* (*Basidiomycota*), are considered well studied in Europe and used as model group in ecological studies and for conservation. Such broad interest, including widespread sampling and DNA based taxonomic revisions, is rapidly transforming our basic understanding of polypore diversity and natural history. We integrated over 40,000 historical and modern records of polypores in Estonia (hemiboreal Europe), revealing 227 species, and including *Polyporus submelanopus* and *P. ulleungus* as novelties for Europe. Taxonomic and conservation problems were distinguished for 13 unresolved subgroups. The estimated species pool exceeds 260 species in Estonia, including at least 20 likely undescribed species (here documented as distinct DNA lineages related to accepted species in, e.g., *Ceriporia, Coltricia*, *Physisporinus*, *Sidera* and *Sistotrema*). Four broad ecological patterns are described: (1) polypore assemblage organization in natural forests follows major soil and tree-composition gradients; (2) landscape-scale polypore diversity homogenizes due to draining of peatland forests and reduction of nemoral broad-leaved trees (wooded meadows and parks buffer the latter); (3) species having parasitic or brown-rot life-strategies are more substrate-specific; and (4) assemblage differences among woody substrates reveal habitat management priorities. Our update reveals extensive overlap of polypore biota throughout North Europe. We estimate that in Estonia, the biota experienced ca. 3–5% species turnover during the twentieth century, but exotic species remain rare and have not attained key functions in natural ecosystems. We encourage new regional syntheses on long studied fungal groups to obtain landscape-scale understanding of species pools, and for elaborating fungal indicators for biodiversity assessments.

**Supplementary Information:**

The online version contains supplementary material available at 10.1186/s43008-020-00050-y.

## INTRODUCTION

The fact that global biodiversity trends are assessed almost without a fungal perspective (e.g., Butchart et al. 2010, IPBES 2018) calls into question how we should integrate scattered mycological knowledge. Historically, regional checklists of fungal biotas have served such aims (e.g., Senn-Irlet et al. 2007), but the rapid advancement of molecular methods and mass data accumulating from ecological assemblage studies challenge such integration (e.g., Peay 2014, Thomson et al. 2018). Thus, molecular biodiversity research is searching its way out of slow nomenclatural procedures (Hibbett 2016); for example, through a concept of species hypothesis based on DNA barcoding (Kõljalg et al. 2013). This causes accumulation of ‘dark taxa’ that lack names or even physical specimens, which cannot currently be used in conventional taxonomy or conservation (Ryberg & Nilsson 2018). For ecological research programs and environmental management, taxonomic and nomenclatural revisions can be too dynamic or impractical, such as when new species are described without morphologically distinct characters (e.g., Korhonen et al. 2018). As a consequence, ecological studies remain taxonomically heterogeneous, often simplified or of unknown quality (Bortolus 2008, Vink et al. 2012), and may omit taxa of critical conservation importance (e.g., rare undescribed species). Taxonomic descriptions, in turn, include only very basic ecological data and seldom report population- and ecosystem-scale context (Durkin et al. 2020). Conservationists have responded with calls to transform taxonomically accepted species lists into special conservation lists to resolve the administrative problem of taxonomic instability (Mace 2004).

With a broader aim to reintegrate disciplines for monitoring fungal diversity, this study provides a new regional synthesis of polyporous fungi (*Agaricomycetes*: *Basidiomycota*; hereafter: polypores) – a conspicuous and well-studied fungal morphogroup. Polypores are distinguished based on poroid hymenophore and mostly lignicolous lifestyle; they inhabit forests on all continents. In recent overviews for Europe and North-America, the number of polypore species was assessed at 400 and 492, accordingly (Zhou et al. 2016, Ryvarden & Melo 2017). Historically, all polypores were included into a common family, *Polyporaceae*, within the order *Aphyllophorales* (Fries 1874). This higher classification based on basidiome morphology was refined by several mycologists in the twentieth century, most notably by Singer (1944), Donk (1948, 1964, 1971), and Jülich (1981), but has been largely rejected since the introduction of molecular systematics. The name *Polyporales* now only refers to one of at least 12 orders within *Agaricomycetes* that include fungi with polyporoid basidiomes (Hibbett et al. 2014). Polyporous ‘morphogenera’ are increasingly replaced by molecularly supported clades that may be closely related to, or even comprise, non-polyporoid fungi (e.g., Miettinen et al. 2012; Runnel et al. 2019). Molecular data have also revealed extensive undescribed species diversity, including morphologically indistinguishable (cryptic) taxa (e.g., Korhonen et al. 2018). Despite these changes in taxonomy and systematics, polypores continue to be treated as a morphogroup in local and regional studies (e.g., Dai 2012, Zhou et al. 2016, Ryvarden & Melo 2017), and in ecological and conservation research. The reasons for that include acceptance by conservationists and educational values.

Functional significance is a major reason why polypores remain a distinct object of research, especially in the fields of forest ecology and conservation. These fungi constitute important decayers, specifically of the huge woody biomass and its lignin component in forests (Floudas et al. 2012). Their mycelia and basidiomes attached to wood provide forage or microhabitat for diverse assemblages of saproxylic invertebrates (e.g., Birkemoe et al. 2018). A subset of polypore species parasitize live trees, some bearing significant economic and social costs for production forestry and arboriculture through root-, butt- and heart-rots (Schwarze et al. 2013). Ecologically, however, heart-rots are key processes in the formation of tree cavities supporting forest fauna (Remm & Lõhmus 2011), while root- and butt-rots promote tree uprooting and trunk breakage (Honkaniemi et al. 2017) that create diverse microhabitats in forests. Several polypore genera include mostly mycorrhizal species, some of which form basidiomes on dead wood (e.g., among *Sistrotrema*; Nilsson et al. 2006, Di Marino et al. 2008). Polypores are best studied in North and Central Europe where intensive forest management has been threatening their diversity – this has facilitated their use for assessing forest conservation values and planning the management (Junninen & Komonen 2011, Halme et al. 2017). Linked with these practical issues has been theoretical interest in polypores as model taxa for metapopulation and assemblage models applicable to dynamic habitat patches (e.g., Ovaskainen et al. 2010; Ramiadantsoa et al. 2018).

To explore the perspectives of this flagship group for fungal diversity assessment, we synthesize diverse information from Estonia – a North European country in the hemiboreal (boreo-nemoral) vegetation zone. The first reliable data on Estonian polypore biota were published in the overview by Dietrich (1856, 1859). Local surveys, with an emphasis on (forest) pathology, were initiated by Elmar Lepik (Leppik) in the late 1920s; he also re-checked and summarized the previously collected material from Estonia (e.g., Lepik 1931, 1940). The forest pathology research direction soon focussed on a few economically significant taxa: *Heterobasidion* species causing butt-rots in conifers (e.g., Karu 1953, Hanso & Hanso 1999) and *Phellinus tremulae* causing heart-rot in European aspen (*Populus tremula*) (reviewed by Tamm 2000). A wider research perspective on polypores, accompanied with taxonomic work, was developed in the second half of the twentieth century by Erast Parmasto (Parmasto 2012). Parmasto (2004) published a monograph that quantitatively summarized all the distribution data on the 211 species then known, their main habitat types and host trees. In the 1990s, Parmasto focused on species sensitive to loss of old-growth forests (Parmasto & Parmasto 1997, Parmasto 2001); this research line has been recently re-assessed based on ecological sampling (e.g., Runnel & Lõhmus 2017). Overall, there has been a large increase in polypore data since 2004 from ecological studies, including the development and testing of the survey methods (Runnel et al. 2015, Lõhmus et al. 2018a). Also species’ distribution mapping has continued, notably through monitoring protected species and in protected areas. However, this new knowledge has remained scattered among projects, and the historical data have not been taxonomically updated.

Our synthesis of the diversity and ecology of Estonian polypores serves three broad aims: (1) We characterize the country-scale species pool in a regional perspective, including taxonomic uncertainties. We do not omit unresolved material; instead, we combine and present molecular phylogenies and habitat data of ‘difficult’ specimens to address the primary aim of describing (full) biodiversity. (2) By critically comparing the updated checklist with Parmasto (2004), we distinguish actual long-term changes in the biota from the advancement of knowledge. And (3) we pool all ecological data to quantitatively analyse compositional similarity of Estonian polypore assemblages and niche characteristics of species. At the ecosystem scale, we assess correspondence between polypore assemblages and the habitat type, specifically in relation to soil conditions, tree composition and stand age. This addresses the ‘Cajanderian’ approach to boreal forest typology, which is based on stable site types rather than temporary conditions (e.g., Frey 1973, Lahti & Väisänen 1987). The practical importance of our ecological analyses is to provide a basis for land-cover or substrate-type proxies for conserving polypore diversity (termed ‘coarse-filter’ and ‘mesofilter’ approaches in conservation biology, respectively; Hunter 2005, Cushman et al. 2008).

## MATERIAL AND METHODS

### Study region and ecosystems

Estonia has a total land area 45,339 km^2^, of which ca. 10% encompasses its western archipelago in the Baltic Sea. The country is situated in the European hemiboreal vegetation zone (Ahti et al. 1968); the natural land cover in the absence of human impact would comprise ca. 85% forest, 8% open wetlands and 5% lakes (Laasimer 1965). The mean air temperature is 17 °C in July and -4 °C in January and the average precipitation is 600–700 mm yr^− 1^. The topography is mostly of glacial origin. Lowlands (post-glacial flooded plains reaching less than 50 m above current sea level) cover nearly half of the territory, and are the dominant land-forms in West-Estonia. The bases of two erosional and three accumulative uplands are 75–100 m above sea level; four of these uplands are in southern Estonia.

Western and eastern Estonia are separated by a borderline of post-glacial landscape history, climate conditions, and land-use patterns (Ahti et al. 1968; Raukas et al. 2004). The last ice sheet retreated ca. five thousand years earlier in the east (Raukas et al. 2004), which now has a more continental climate with isotherm differences up to 4–5 °C compared with western Estonia (Jõgi & Tarand 1995). This border can be also recognised in the distribution of biodiversity, such as plants (Laasimer 1965) and epiphytic lichens (Jüriado et al. 2003).

Forests, the main ecosystem hosting polypores, currently cover 51% of Estonia but, after a long history of land use, only 2% of this is old natural stands (Raudsaar et al. 2018). Forest conversion to agriculture reached its maximum by the 1930s when ca. one-third of the country had woodland cover (Meikar & Uri 2000). Subsequent afforestation mostly took place due to the abandonment of small agricultural fields and wetland drainage for forestry. Timber harvest intensities were relatively low in the second half of the twentieth century, but rapidly increased after the country regained independence: from 2 to 3 million m^3^ in 1991–1993 to 10–12 million m^3^ in 2000–2001 where the volume stabilized, after a temporary decline, since 2011. In the same period, strictly protected forest reserves were expanded from ca. 3 to 13% of forest land (Lõhmus et al. 2004, Raudsaar et al. 2018). The forest management has been based on native tree species and, to a significant extent, on natural regeneration (‘semi-natural forestry’), but following the even-aged (clear-cutting based) silvicultural system and including planting (mostly conifers), thinning, and artificial drainage. Such a mixture of approaches maintained commercial forests in a relatively favourable state for wood-inhabiting species (Lõhmus et al. 2016, Runnel & Lõhmus 2017). However, recent developments to lower rotation age, increase cut-block size, subsidized planting, and (in private forests) ditching threaten forest biodiversity in a longer perspective (e.g., Lõhmus et al. 2018b).

Based upon edaphic and hydrological factors, nine natural and two anthropogenic forest site type groups (drained peatlands; reclaimed areas), comprising at least 27 forest site types, are distinguished for the practical planning and monitoring of Estonian forests (Lõhmus 1984, Raudsaar et al. 2018; Additional file 1). Common natural site type groups are meso-eutrophic (27%; usually Norway spruce *Picea abies* mixtures with deciduous trees), dry boreal (23% of forest land; most dominated by Scots pine, *Pinus sylvestris*), eutrophic paludifying (16%; mostly birch *Betula* spp., often in mixtures with *P. sylvestris*), and eutrophic boreo-nemoral forests (10%; typically *Betula* spp., *Populus tremula*, and grey alder *Alnus incana*). The dominant anthropogenic forests are drained peatland forests (14%; mostly *Betula* spp. and *P. sylvestris*). All the main forest trees are native; 31% of forest area is dominated by *P. sylvestris*, 30% by *Betula* spp., 19% by *P. abies*, 9% by *Alnus incana* and 6% by *P. tremula* (Raudsaar et al. 2018). Stands of exotic trees comprise 0.1% of forest land. Over 25% of the forest land has been drained and over 300,000 ha planted, but there are few intensive plantations and stands usually consist of more than one (most often three) tree species.

The main secondary habitats for polypores are semi-natural and urban areas with sparse tree cover. Of these, most traditional wooded meadows were lost during the twentieth century due to the re-organization of agriculture; only < 10,000 ha remain (Sammul et al. 2008). Compared with Western Europe, the Estonian agricultural landscapes still retain significant areas with natural components such as scattered tree rows and single trees (e.g., Kikas et al. 2018). Biodiversity hotspots in the countryside include rural parks that may have dead wood amounts comparable with those in production forests (e.g., Lõhmus & Liira 2013), and riparian zones that contain specific habitats (such as large *Salix* trees) rarely found in forests. Finally, ca. 2% of Estonian land cover comprises human settlements, often with a significant proportion of green space and trees. A distinct polypore habitat feature of the green space is a diverse mixture of exotic tree species, planted as ornamental species or sometimes as tree collections. Tallinn alone (excluding its botanical garden) hosts 449 exotic species in addition to the 31 native species of trees (Sander et al. 2003).

### Estonian polypore data

The Estonian polypore data used includes *ca* 40,500 basidiome records (Table 1). A ‘record’ refers to collected specimens or archived observations, usually at the level of one distinct substrate unit (e.g., a single fallen trunk). About 10% of records – such as some historical species lists and ecological studies (e.g., Lõhmus 2011) – refer to occurrences at the scale of a forest stand. The specimens we collected are deposited in the fungaria of Tartu University (TU) and the Estonian University of Life Sciences (TAAM); all these records, together with their molecular DNA data and occasional photographs, are archived in the PlutoF database (Abarenkov et al. 2010). At the time of compiling of this study, the molecular data (mostly ITS sequences; in a minority of cases additionally LSU sequences) were available for 3% of all records (Table 1).

**Table 1:** Main sources of the Estonian polypore data

Data source or sampling design	No. of records (sequences)^a^	Studied ecosystems	Publications
I. Historical data until 2004	13,249 (48)	All	Parmasto 2004
IIa. Systematic sampling in a 4-km^2^ forest landscape in E Estonia, 2008–2009	3560 (3)	All forest land; mostly eutrophic and meso-eutrophic mixed sites	Lõhmus 2011
IIb. Standard surveys in 30 2-ha plots and their surroundings in SW Estonia, 2013	2393 (122)	*Pinus sylvestris* dominated drained peatland forests	Runnel et al. 2015 10.15156/BIO/786358
IIc. Standard surveys in 144 2-ha plots, 2005–2016	17,012 (334)	Forests and clear-cuts of various types, except of bog and drained wetland types	Runnel & Lõhmus 2017 10.15156/BIO/786363 10.15156/BIO/786357
IId. Fallen retention trees in 48 clear-cuts in mainland, 2010–2011	259 (19)	Sites on mineral soils	Runnel et al. 2013
IIIa. Casual collections after 2004	3020 (631)	All	PlutoF database
IIIb. Surveys of 27 species in protected areas, 2015–2016	922 (89)	All	PlutoF database (partly)
Total	40,415 (1246)		

^a^ no. of sequences deposited in the PlutoF database (Abarenkov et al. 2010)

**Fig. 1:**
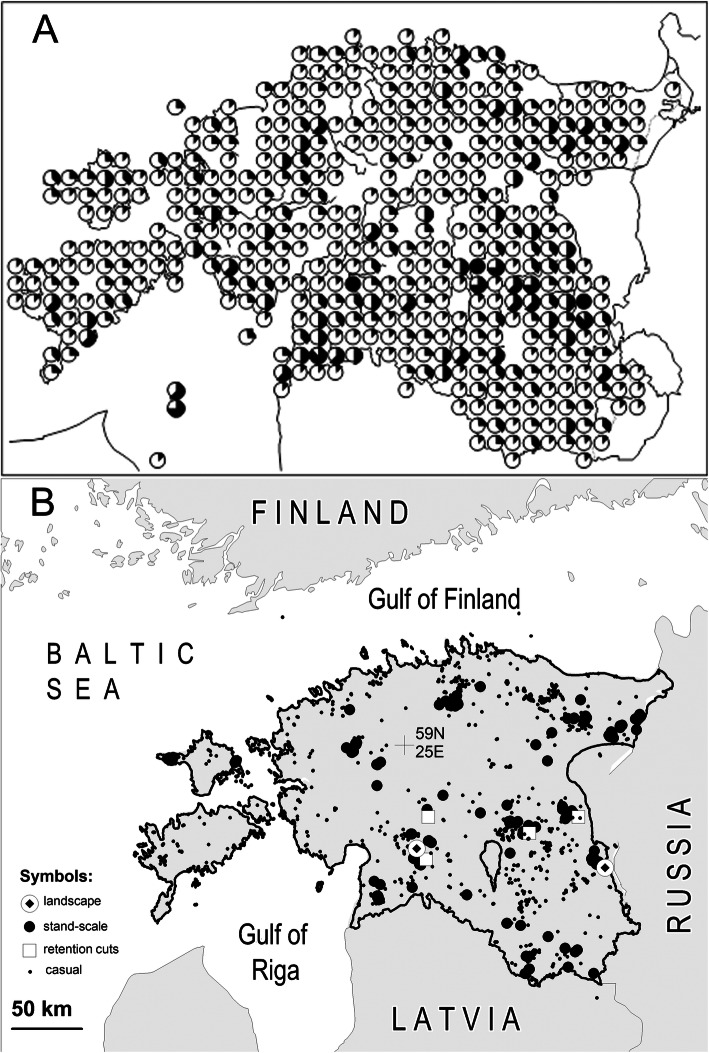
Distribution of the Estonian polypore datasets included in this study. **a** Data until 2004: relative no. of species of the total species pool on the 10 × 10 km UTM-grid as reported by Parmasto (2004). **b** Systematic surveys and casual records in 2005–2018. ‘Landscape’ surveys refer to intensive sampling of the Soomaa area in the west (Runnel et al. 2015 and unpubl.) and Aravu area in the east (Lõhmus 2011). ‘Stand-scale’ surveys are standard-effort surveys in 2-ha plots (Lõhmus et al. 2018a). ‘Retention-cut’ sampling was from selected trunks (Runnel et al. 2013). Casual records are all other records and observations extracted from PlutoF database, 8 November 2018. Graph (A) reproduced with the permission of the Estonian University of Life Sciences, Tartu

The material comprised three methodologically distinct parts.

(I) One-third of the material were all records until 2004, which were originally summarized by Parmasto (2004). These are mostly specimens collected during casual surveys by Parmasto and his colleagues in the period 1950–2004, and a critical revision of all older collections. The material has been sampled throughout the country, although some regions (such as eastern and south-western Estonia) have been more intensively covered (Fig. 1A). Parmasto (2004) admits paying more attention to *Phellinus* (*sensu* *lato*) and old-forest fungi; a re-analysis of the whole dataset by Lõhmus (2009) suggested a more general bias (compared with frequencies in nature) toward easily recognizable species with perennial basidiomes. A preference to visit certain biodiversity ‘hotspots’ (such as protected areas, some maritime islands and certain city parks) is also obvious in the location data. For the current study, most original specimens of poorly identifiable rare species (see Lõhmus 2009 and under “Difficult species” below) were morphologically re-checked and, by necessity, sequenced (Table 1).
(II) Fifty-seven percent of all records were obtained from systematic surveys of polypore assemblages by K.R and A.L in 2005–16. These surveys have been planned and (mostly) published to address questions of forest ecology and conservation (Table 1). Accordingly, this material represents most Estonian forest ecosystems, although it is geographically biased toward mainland Estonia, especially southern, eastern, and north-eastern parts of the country (Fig. 1B). The surveys were performed in the top basidiome production season (September–October), with efforts to record all species either at the habitat patch or substrate scale (to analyse also species absences) along with detailed descriptions of the habitats and substrates. The substrate descriptions have routinely included tree species, condition, diameter, and decay stage (five classes, I–V, according to Renvall 1995). About 15% of the field observations are supported by collections, focusing on basidiomes that could not be reliably identified in the field, represented poorly studied taxa, or atypical substrates (Runnel et al. 2014, Lõhmus et al. 2018a). The collected basidiomes have all been inspected microscopically and ca. 20% of the specimens have been sequenced (Table 1).

Three field protocols were followed in the systematic surveys. The *main set* of surveys (Table 1: IIb–IIc; 48% of all records) followed a fixed-area-fixed-effort survey protocol, as presented and analysed for bias by Lõhmus et al. (2018a). Each survey was carried out during 4 h in a precisely delineated 2-ha plot by a single observer (the plots listed in Additional file 2). For each species in each plot, substrates of the first ten records were described in detail. Up to 150 such records per plot could be obtained within the 4 h. A less thorough method was used in an East Estonian *forest landscape* study (Table 1: IIa), where all forest stands in a 4-km^2^ area were sampled by adjusting survey time with stand area (range 0.1–7 ha; see Lõhmus 2011 for details). For most species, one substrate type in one stand comprised one record, but rare and threatened species were recorded at the scale of individual substrate items. Finally, a small study on *retention trees* in four Estonian regions recorded all species at the scale of individual tree trunks (Runnel et al. 2013; Table 1: IId).

III Post-2004 casual records comprise 10% of all records, from two sources (Table 1). The majority are specimen and observation data as extracted on 8 November 2018 from the PlutoF database (Abarenkov et al. 2010). These data originate from casual surveys similar to Parmasto’s (2004) material from professional and, increasingly, amateur mycologists all over Estonia (Fig. 1b). All the observations obtained from the database were quality-scanned, and doubtful identifications were discarded. We additionally included 922 observations of 27 easily identifiable protected, rare or old-forest indicator species (full list is available upon request) during publicly funded fungal surveys by Indrek Sell in two protected areas in mainland Estonia: the Soomaa National Park in 2015 and the Muraka Nature Reserve in 2016.

#### Data processing

##### Updating species list and documenting taxonomic uncertainties

For ecological analyses, the set of casual records included in this paper are as of 8 November 2018. However, Table 2 has been updated based on casual collection data (Table 1: IIIa) as of 20 July 2019, with the records of *Amylocystis lapponica* updated according to Runnel et al. (2020), and *Inonotus ulmicola* and *Spongipellis spumea* including the observations by Pau (2018). *Phellinus igniarius* sensu stricto is defined as all species records from *Salix* spp.

**Table 2:** Estonian polypore species, their voucher specimens in fungaria, no. of records by sources (I: historical data up to 2004; II: systematic sampling; III: casual collections), habitats, and national Red-List status (Category and Criteria). Habitat data denote presence by: forest successional stage (E, early-successional; M, mid-successional; L, late-successional forests), host tree species (S, *Picea abies*; P, *Pinus sylvestris*; A, *Populus tremula*; B, *Betula* spp.; D, other deciduous species, O, ornamental conifers), woody substrate (C, coarse downed deadwood; F, fine downed or standing deadwood; Sn, snags and stumps; L, live trees), and decay stage (E, early; M, medium; L, late). For species with ≥25 records from systematic sampling, the habitat summary is given as % of species records in the systematic sample

	Species^**1**^		Voucher	No. of records			Succ. stage	Tree species	Subst. type	Decay stage	Cat.	Crit.
				I	II	III						
^p^	*Abortiporus biennis*	(Bull.) Singer	TU104564	2	0	4		D	L		VU	D1
^m^	*Albatrellus citrinus*	Ryman	TU106597	16	16	10	ML		G		LC	
^m^	*Albatrellus confluens*	(Alb. & Schwein.) Kotl. & Pouzar	TU106802	24	1	1	L		G		DD	
^m^	*Albatrellus ovinus*	(Schaeff.) Kotl. & Pouzar	TU118663	69	51	11	51M49L		G		LC	
^m^	*Albatrellus subrubescens*	(Murrill) Pouzar	TU106803	14	0	2			G		DD	
	*Amylocystis lapponica*	(Romell) Bondartsev & Singer ex Singer	TAAM189465	1	1	59	L	S	C	EML	CR	D1
	*Anomoloma albolutescens*	(Romell) Niemelä & K.H. Larss.	TAAM174473	1	0	0					EN	D1
	*Anomoloma myceliosum*	(Peck) Niemelä & K.H. Larss.	TU101934	5	26	1	65M35L	27S73P	27C73F	42E38M19L	VU	D1
	*Anomoporia bombycina*	(Fr.) Pouzar	TU111116	6	0	3		SP	C		NT	D1
	*Antrodia cretacea*	K. Runnel, V. Spirin & A. Lõhmus	TU121005	6	6	7	EL	S	CSn	EML	EN	B2ab(ii,iii); C2a(i)
	*Antrodia heteromorpha*	(Fr.) Donk	TAAM001039	4	0	0					RE	
	*Antrodia leucaena*	Y.C. Dai & Niemelä	TU129577	1	7	7	EML	A	CF	EM	VU	C1
	*Antrodia macra*	(Sommerf.) Niemelä	TU129573	9	4	3	EM	DA	F	EM	DD	
	*Antrodia mellita*	Niemelä & Penttillä	TU114649	7	0	7		A	C		EN	D1
	*Antrodia piceata*	K. Runnel, V. Spirin & J. Vlasák	TU129574	4	13	19	L	S	C	EML	EN	B1ab(iv,v)+2ab(iv,v); C2a(i)
	*Antrodia pulvinascens*	(Pilát) Niemelä	TU117272	12	2	42	E	A	CFSn	M	VU	D1
	*Antrodia ramentacea*	(Berk. & Broome) Donk	TU122933	3	3	3	M	P	F	EM	DD	
	*Antrodia serialis*	(Fr.) Donk	TU120464	>100	>100	25	24E33M44L	95S5P1D	72C11Sn17F	32E59M9L	LC	
	*Antrodia sinuosa*	(Fr.) P. Karst.	TU117300	>100	>100	29	17E49M34L	38S61P1B0A	72C2Sn26F	22E57M21L	LC	
	*Antrodia xantha*	(Fr.) Ryvarden	TU117274	>100	>100	17	13E37M50L	10S89P1D	73C6Sn21F	27E56M18L	LC	
	*Antrodiella citrinella*	Niemelä & Ryvarden	TU117326	2	30	13	10E17M73L	83S3P7B7A	90C3Sn7F	10E30M60L	LC	
	*Antrodiella faginea*	Vampola & Pouzar	TU129240	3	20	4	EML	PDBA	CSnF	EML	LC	
	*Antrodiella niemelaei*	Vampola & Vlasák	TU130078	2	1	3	E	D	F	L	VU	D1
	*Antrodiella pallescens*	(Pilát) Niemelä & Miettinen	TU117329	74	>100	9	12E72M16L	11D86B3A	54C15Sn31F	7E53M40L	LC	
	*Antrodiella parasitica*	Vampola	TAAM164505	1	0	0					EN	D1
	*Antrodiella romellii*	(Donk) Niemelä	TU129193	19	47	6	20E73M7L	60D33B7A	9C2Sn88F	48E40M12L	LC	
	*Antrodiella serpula*	(P. Karst.) Spirin & Niemelä	TU115534	72	78	19	5E65M29L	87D10B3A	23C37Sn40F	23E54M23L	LC	
	*Aporpium canescens*	(P. Karst.) Bondartsev & Singer ex Singer	TU129596	23	39	13	5E38M57L	3S5D41B51A	73C19Sn8F	24E38M38L	LC	
	*Aporpium macroporum*	T. Niemelä, V. Spirin & O. Miettinen	TU129583	2	6	17	EML	A	CSn	EM	VU	C1
^p?^	*Aurantiporus croceus*	(Pers.) Murrill	TU111103	2	0	6		D	L		CR	D1
^p?^	*Aurantiporus fissilis*	(Berk. & M.A. Curtis) H. Jahn ex Ryvarden	TU117130	14	7	2	ML	DA	CF	EML	NT	D1
	*Aurantiporus priscus*	Niemelä, Miettinen & Manninen	TAAM199826	3	0	0					CR	C2a(i); D1
	*Bjerkandera adusta*	(Willd.) P. Karst.	TU118559	>100	>100	35	34E42M24L	4S34D35B35A	44C32Sn22F1L	52E40M8L	LC	
	*Bjerkandera fumosa*	(Pers.) P. Karst.	TAAM183439	40	4	4	M	DB	CSnF	EM	LC	
^m^	*Boletopsis grisea*	(Peck) Bondartsev & Singer	TU117299	15	0	13			G		VU	D1
^m^	*Boletopsis leucomelaena*	(Pers.) Fayod	TU120100	4	1	3	L		G		EN	C2a(i)
	*Botryodontia millavensis*	(Bourdot & Galzin) Duhem & H. Michel	TAAM201266	18	0	22		O	FL		VU	D1
	*Byssoporia terrestris*	(DC.) M.J. Larsen & Zak	TU130449	1	7	0	EML	SPA	CF	EML	VU	D1
	*Ceriporia aurantiocarnescens*	(Henn.) M. Pieri & B. Rivoire	TU122039	0	2	3	M	DA	C	M	DD	
	*Ceriporia bresadolae*	(Bourdot & Galzin) Donk	TU122499	3	0	1		P		M	EN	D1
	*Ceriporia excelsa*	(S. Lundell) Parmasto	TU117255	18	32	8	53E31M16L	13D66B22A	75C3Sn22F	3E65M32L	LC	
	*Ceriporia purpurea*	(Fr.) Donk	TU115545	20	11	9	EML	DBA	CSnF	EML	LC	
	*Ceriporia reticulata*	(Hoffm.) Domanski	TU121840	21	51	5	40E36M24L	2P48D30B20A	24C2Sn74F	22E58M20L	LC	
	*Ceriporia tarda*	(Berk.) Ginns	TAAM196177	1	0	8		S			CR	D1
	*Ceriporia torpida*	Spirin & Miettinen		0	0	1		A			DD	
	*Ceriporia viridans*	(Berk. & Broome) Donk	TU129216	34	41	9	48E28M25L	3P18D53B28A	45C8Sn48F	3E70M27L	LC	
	*Ceriporiopsis aneirina*	(Sommerf.) Domanski	TU117256	37	70	28	36E29M36L	100A	69C1Sn30F	61E37M2L	LC	
	*Ceriporiopsis pseudogilvescens*	(Pilát) Niemelä & Kinnunen	TU129597	2	14	11	ML	DA	CSnF	EML	LC	
	*Ceriporiopsis resinascens*	(Romell) Domanski	TU115564	19	2	1	EL	DA	CF	M	LC	
	*Cerrena unicolor*	(Bull.) Murrill	TU101682	>100	>100	17	55E32M13L	4D92B4A	28C41Sn30F	28E51M21L	LC	
	*Cinereomyces lindbladii*	(Berk.) Jülich	TU117291	28	>100	22	53E25M22L	44S32P6D18B	63C1Sn36F	7E52M41L	LC	
	*Climacocystis borealis*	(Fr.) Kotl. & Pouzar	TU118900	>100	42	29	26E17M57L	100S	31C69Sn	54E36M10L	LC	
^m^	*Coltricia cinnamomea*	(Jacq.) Murrill	TU106861	0	0	6			G		VU	D1
^m^	*Coltricia confluens*	P.-J. Keizer	TAAM181460	0	0	2			G		NE	
^m^	*Coltricia perennis*	(L.) Murrill	TU120468	>100	13	9	EML		G		LC	
	*Daedalea quercina*	(L.) Pers.	TU106561	>100	0	18		D	CL		LC	
	*Daedaleopsis confragosa*	(Bolton) J. Schröt.	TU118931	87	49	42	14E71M14L	90D10B	16C39Sn43F2L	47E46M7L	LC	
	*Datronia mollis*	(Sommerf.) Donk	TU109290	>100	>100	21	21E49M29L	26D34B40A	39C3Sn58F	51E42M7L	LC	
	*Dichomitus campestris*	(Quel.) Domanski & Orlicz	TU117217	12	8	26	M	D	SnF	EM	NT	D1
	*Dichomitus squalens*	(P. Karst.) D.A. Reid	TU121329	6	4	0	EML	SP	CF	EM	EN	C2a(i)
	*Diplomitoporus crustulinus*	(Bres.) Domanski	TAAM134247	1	0	0					RE	
	*Diplomitoporus flavescens*	(Bres.) Domanski	TU101542	52	>100	48	8E90M3L	1S99P	11C66Sn23F	83E17M	LC	
	*Fiboporia gossypium*	(Speg.) Parmasto	TU117247	8	8	14	EML	SP	CF	EML	VU	D1
	*Fibroporia norrlandica*	(Berglund & Ryvarden) Niemelä	TU129622	0	12	0	EM	SPB	CF	EM	LC	
	*Fibroporia vaillantii*	Parmasto	TAAM184863	12	1	1	E	PO	F	L	VU	D1
^p^	*Fistulina hepatica*	(Schaeff.) With.	TU118753	86	0	11		D	L		NT	D1
	*Fomes fomentarius*	(L.) Fr.	TU117322	>100	>100	55	17E58M25L	5D93B2A	45C37Sn18F	38E48M14L	LC	
	*Fomitopsis pinicola*	(Sw.) P. Karst.	TU117240	>100	>100	>100	16E55M28L	49S15P8D24B4A	52C37Sn10F	41E50M9L	LC	
	*Fomitopsis rosea*	(Alb. & Schwein.) P. Karst.	TU118898	60	>100	>100	1E16M84L	99S1P	93C1Sn6F	28E63M7L	NT	A3(c)
	*Funalia trogii*	(Berk.) Bondartsev & Singer	TAAM202749	20	73	8	83E10M6L	2D4B93A	50C317F2L	41E57M2L	NT	A3(c)
	*Ganoderma applanatum*	(Pers.) Pat.	TU129233	>100	>100	54	37E30M34L	1S20D41B37A	51C46Sn3F1L	12E32M54L	LC	
	*Ganoderma carnosum*	Pat.	TAAM126866	1	0	0					NA	
	*Ganoderma lucidum*	(Curtis) P. Karst.	TU129603	40	25	24	44E52M4L	20S20D56B4A	28C68Sn4F	9M91L	LC	
	*Gelatoporia subvermispora*	(Pilát) Niemelä	TU122080	1	6	6	EML	SDBA	CF	ML	NT	D1
	*Gloeophyllum abietinum*	(Bull.) P. Karst.	TU111350	48	12	10	EML	S	CF	EM	NT	C1
	*Gloeophyllum odoratum*	(Wulfen) Imazeki	TU118351	>100	>100	31	74E13M14L	93S4P1D	16C84Sn	5E52M43L	LC	
	*Gloeophyllum sepiarium*	(Wulfen) P. Karst.	TU106410	>100	>100	25	76E16M8L	83S9P1B7A	38C14Sn48F	48E46M5L	LC	
	*Gloeophyllum trabeum*	(Pers.) Murrill	TU120451	13	10	4	E	DBA	CSnF	EM	LC	
	*Gloeoporus dichrous*	(Fr.) Bres.	TU114774	42	51	4	16E70M14L	2S2D92B4A	43C20Sn37F	27E49M24L	LC	
	*Gloeoporus pannocinctus*	(Romell) J. Erikss.	TU117298	46	51	12	4E53M43L	2S35D55B8A	84C10Sn4F2L	30E38M32L	LC	
^p^	*Grifola frondosa*	(Dicks.) Gray	TU120007	8	0	10		DO	L		CR	D1
	*Hapalopilus aurantiacus*	(Rostk.) Bondartsev	TU129768	5	3	3	E	SP	CSn	EM	EN	D1
	*Hapalopilus ochraceolateritius*	(Bondartsev) Bondartsev & Singer	TU121752	4	2	3	E	SP	CSn	M	EN	D1
	*Hapalopilus rutilans*	(Pers.) Murrill	TU118885	78	51	23	6E86M8L	2S18D80B	14C14Sn73F	22E62M16L	LC	
	*Haploporus tuberculosus*	(Fr.) Niemelä & Y.C. Dai	TAAM201265	1	0	1		D			CR	D1
^p^	*Heterobasidion annosum*	(Fr.) Bref.	TAAM201141	20	2	19	EM	PB	CSn	EL	LC	
^p^	*Heterobasidion parviporum*	Niemelä & Korhonen	TU118616	>100	>100	27	12E34M55L	98S2P1B	77C20Sn3F1L	34E50M16L	LC	
	*Hyphodontia flavipora*	(Berk. & M.A. Curtis ex Cooke) Sheng H. Wu	TU129751	2	7	2	ML	DBA	CF	EM	NT	D1
	*Hyphodontia latitans*	(Bourdot & Galzin) E. Langer	TU129697	1	7	4	ML	DBA	CF	EML	EN	B1ab(iv,v)+2ab(iv,v); C2a(i); D1
	*Hyphodontia radula*	(Schrader) E. Langer & Vesterholt	TU129685	8	>100	6	7E68M25L	1S1P40D55B3A	34C4Sn62F1L	25E57M18L	LC	
	*Hyphodontia paradoxa*	(Fr.) Langer & Vesterh.	TU111288	33	26	4	15E50M35L	54D38B8A	4C12Sn85F	15E66M19L	LC	
	*Inonotopsis subiculosa*	(Peck) Parmasto	TAAM058545	1	0	0					RE	
^p^	*Inonotus dryadeus*	(Pers.) Murrill		1	0	0					RE	
^p^	*Inonotus dryophilus*	(Berk.) Murrill	TAAM196870	2	0	2		D	L		CR	D1
^p^	*Inonotus obliquus*	(Ach. ex Pers.) Pilát	TU120209	>100	>100	24	2E71M27L	6D93B1A	6C19Sn2F73L	75E20M6L	LC	
	*Inonotus radiatus*	(Sowerby) P. Karst.	TU118763	>100	>100	35	14E62M24L	84D16B1A	27C52Sn18F2L	51E42M7L	LC	
	*Inonotus rheades*	(Pers.) P. Karst.	TAAM171920	39	1	12	M	A	F	E	NT	D1, C1
^p^	*Inonotus ulmicola*	Corfixen	TAAM178664	5	0	45		D	L		NT	D1
	*Irpex lacteus*	(Fr.) Fr.	TU121351	14	11	4	E	DB	F	EML	LC	
	*Ischnoderma benzoinum*	(Wahlenb.) P. Karst.	TU120112	>100	>100	36	11E35M54L	78S22P	72C21Sn7F	32E50M18L	LC	
	*Junghuhnia autumnale*	Spirin, Zmitr. & Malysheva	TU129604	0	6	1	ML	DA	CF	E	VU	D1
	*Junghuhnia collabens*	(Fr.) Ryvarden	TU117284	10	40	43	8M93L	98S3P	95C3Sn3F	8E38M55L	NT	C1
	*Junghuhnia fimbriatella*	(Peck) Ryvarden	TU117288	2	1	8	L	SDA	C	L	EN	D1
	*Junghuhnia lacera*	(P. Karst.) Niemelä & Kinnunen	TU117302	1	6	2	EML	DBA	CF	EML	DD	
	*Junghuhnia luteoalba*	(P. Karst.) Ryvarden	TU122833	14	>100	9	26E67M7L	6S94P	59C5Sn36F	16E70M14L	LC	
	*Junghuhnia nitida*	(Pers.: Fr.) Ryvarden	TU117246	81	>100	14	26E58M17L	1S42D32B26A	13C2Sn85F	22E61M17L	LC	
^p^	*Junghuhnia pseudozilingiana*	(Parmasto) Ryvarden	TU111359	27	24	>100	ML	BA	CSnFL	EM	VU	C1
^p^	*Laetiporus sulphureus*	(Bull.) Murrill	TU118392	>100	5	40	L	DA	CF	EM	LC	
	*Lenzites betulina*	(L.: Fr.) Fr.	TU120080	99	>100	19	59E36M6L	1S6D81B12A	23C15Sn61F	46E47M7L	LC	
	*Leptoporus erubescens*	(Fr.) Bourdot & Galzin	TU117236	6	9	19	EML	100P	CSn	EM	NE	
	*Leptoporus mollis*	(Pers.) Quél.	TU129905	47	35	56	9E45M46L	100S	66C12Sn21F1L	56E44M	LC	
	*Lindtneria trachyspora*	(Bourdot & Galzin) Pilát		5	1	0	L	B	C	L	EN	D1
	*Meruliopsis taxicola*	(Pers.) Bondartsev	TU120635	42	26	14	12E69M19L	4S96P	65C15Sn19F	85E15M	LC	
	*Obba rivulosa*	(Berk. & M.A. Curtis) Miettinen & Rajchenb.	TU121738	0	1	0	M	S	F	M	EN	D1
^p^	*Onnia leporina*	(Fr.) H. Jahn	TU129416	34	4	6	L	S	CSn	E	EN	A2(a); 4(a,b); C1
^p^	*Onnia tomentosa*	(Fr.: Fr.) P. Karst.	TU106685	47	5	26	EML	SA	F	M	LC	
	*Oxyporus corticola*	(Fr.) Ryvarden	TU117341	>100	88	40	17E23M60L	8S5D6B81A	78C9Sn12F1L	48E45M7L	LC	
	*Oxyporus latemarginatus*	(E.J. Durand & Mont.) Donk	TU121210	2	2	1	E	B	Sn	ML	EN	D1
	*Oxyporus obducens*	(Pers.) Donk	TAAM202744	1	0	1		D	F		DD	
^p^	*Oxyporus populinus*	(Schumach.) Donk	TU118657	>100	64	27	2E47M52L	59D34B6A	14C25Sn3F58L	56E33M11L	LC	
	*Oxyporus ravidus*	(Fr.) Bondartsev & Singer	Niemelä 7215	2	0	0					EN	D1
^p^	*Perenniporia medulla-panis*	(Jacq.) Donk	TAAM189567	9	0	3		D	C		EN	D1
	*Perenniporia narymica*	(Pilát) Pouzar	TU128008	0	1	0		B	C	L	NA	
	*Perenniporia subacida*	(Peck) Donk	TU117317	28	20	27	EML	SPDBA	CSnF	EML	NT	D1
	*Perenniporia tenuis*	(Schwein.) Ryvarden	TAAM189637	1	0	0					CR	CR B1a,b(i,v)+2a,b (i,v); C2a(i); D1
^p^	*Phaeolus schweinitzii*	(Fr.) Pat.	TU118304	45	3	14	L	SP	CSnE	M	NT	D1
^p^	*Phellinus alni*	(Bondartsev) Parmasto	TAAM191398	>100	>100	51	4E52M44L	100D	18C19Sn3F60L	71E27M2L	LC	
^p^	*Phellinus chrysoloma*	(Fr.) Donk	TU118916	>100	71	75	30M70L	100S	28C27Sn3F42L	60E38M3L	LC	
^p^	*Phellinus conchatus*	(Pers.) Quél.	TU120539	>100	50	23	14E64M22L	100D	32C30Sn14F24L	46E46M8L	LC	
	*Phellinus ferrugineofuscus*	(P. Karst.) Bourdot	TU117283	35	28	>100	29M71L	100S	93C7F	23E58M19L	NT	C1
	*Phellinus ferruginosus*	(Schrad.) Pat.	TU111127	18	2	29	M	D	CSnF	E	LC	
	*Phellinus hippophaeicola*	H. Jahn	TU128014	0	0	1					NE	
^p^	*Phellinus igniarius*	(L.) Quél.	TAAM191408	>100	21	27	EML	D	CSnFL	EM	LC	
	*Phellinus laevigatus*	(Fr.) Bourdot & Galzin	TU117262	>100	>100	14	6E38M56L	100B	60C8Sn32F1L	32E57M11L	LC	
^p^	*Phellinus lundellii*	Niemelä	TU117270	22	17	5	EML	DB	CSnF	EML	LC	
^p^	*Phellinus nigricans*	(Fr.) P. Karst.	TU122789	>100	>100	17	10E65M25L	100B	20C41Sn9F31L	51E40M9L	LC	
	*Phellinus nigrolimitatus*	(Romell) Bourdot & Galzin	TU117268	45	49	45	4E12M84L	100S	98C2F	6E81M13L	LC	
^p^	*Phellinus pini*	(Brot.) A. Ames	TU109982	>100	>100	>100	32M68L	100P	2C13Sn85L	86E14M	LC	
^p^	*Phellinus populicola*	Niemelä	TU111223	>100	34	84	9E59M32L	100A	9C21Sn71L	70E30M	LC	
^p^	*Phellinus punctatus*	(P. Karst.) Pilát	TU120544	>100	93	27	11E68M22L	96D3B1A	14C44Sn30F12L	49E42M9L	LC	
^p^	*Phellinus robustus*	(P. Karst.) Bourdot & Galzin	TU118766	54	0	7		D	L		LC	
^p^	*Phellinus tremulae*	(Bondartsev) Bondartsev & P.N. Borisov	TAAM191400	>100	>100	44	12E56M32L	100A	12C3Sn2F83L	73E26M2L	NT	A2
^p^	*Phellinus tuberculosus*	(Baumg.) Niemelä	TAAM196106	60	0	2		O	L		LC	
	*Phellinus viticola*	(Schwein.) Donk		1	0	0					RE	
^p^	*Phylloporia ribis*	(Schumach.) Ryvarden	TAAM196444	29	0	21		O	L		LC	
	*Physisporinus sanguinolentus*	(Alb. & Schwein.) Pilát	TU117267	9	>100	9	46E24M30L	36S15P26D18B5A	45C20Sn35F	20E47M33L	LC	
	*Physisporinus vitreus*	(Pers.) P. Karst.	TU122877	51	99	5	42E36M22L	7S1P69D19B3A	37C6Sn57F	25E59M16L	VU	D1
	*Physisporinus undatus*	(Pers.) Donk	TU117254	3	1	2	EML	DS	C	L	NE	
	*Piptoporus betulinus*	(Bull.) P. Karst.	TU118904	>100	>100	56	3E78M19L	100B	17C23Sn59F1L	55E42M3L	LC	
	*Polyporus badius*	(Pers.) Schwein.	TU120199	19	51	46	6E33M61L	2S26D10B62A	76C10Sn14F	15E70M15L	NT	A2
	*Polyporus brumalis*	(Pers.) Fr.	TU129816	79	79	10	81E16M3L	30D68B1A	11C9Sn80F	15E52M33L	LC	
	*Polyporus ciliatus*	Fr.	TU118306	81	91	8	97E2M1L	18D81B1A	7C19Sn74F1L	10E56M34L	LC	
	*Polyporus leptocephalus*	(Jacq.) Fr.	TU106392	>100	40	31	23E45M33L	9D41B50A	44C3Sn53F	55E39M6L	LC	
	*Polyporus melanopus*	(Pers.) Fr.	TU118072	3	0	16		DP	GC		VU	D1
^p^	*Polyporus pseudobetulinus*	(Murashk. ex Pilát) Thorn, Kotir. & Niemelä		1	0	0					RE	
	*Polyporus (Cerioporus) rangiferinus*	(Bolton) Zmitr., Volobuev, I. Parmasto & Bondartseva	TU117400	2	1	7	L	D	GC		DD	
^p^	*Polyporus squamosus*	(Huds.) Fr.	TAAM205584	>100	0	27	L	D	CL	M	LC	
	*Polyporus submelanopus*	H.J. Xue & L.W. Zhou	TAAM185810	1	0	0					NA	
	*Polyporus tubaeformis*	(P. Karst.) Ryvarden & Gilb.	TU114240	8	0	0					VU	D1
	*Polyporus ulleungus*	H. Lee, N.K. Kim & Y.W. Lim	TU129852	0	1	0	M	B	F	M	NE	
^p?^	*Polyporus umbellatus*	(Pers.) Fr.	TU113528	7	0	12			G		EN	D1
	*Porotheleum fimbriatum*	(Pers.) Fr.	TU129702	95	>100	9	29E31M39L	18S3P25D37B17A	26C5Sn69F	21E55M24L	LC	
	*Porpomyces mucidus*	(Pers.) Jülich	TU117331	45	24	9	EML	SPDBA	CSnF	EML	LC	
	*Postia auricoma*	Spirin & Niemelä	TU129235	0	3	1	ML	P	C	EL	VU	D1
	*Postia balsamea*	(Peck) Jülich	TU128005	1	0	1		O	L		DD	
	***Postia caesia sl***			>100	>100	31	11E51M38L	60S7P14D6B13A	43C4Sn53F	40E56M4L	LC	
	*- Postia alni*	Niemelä & Vampola	TU130387									
	*- Postia caesia*	(Schrad.) P. Karst.	TU127228									
	*- Postia cyanescens*	Miettinen	TU130591									
	*- Postia populi*	Miettinen	OM21796									
	*- Postia simulans*	(P. Karst.) Spirin & B. Rivoire	TU129873									
	*Postia ceriflua*	(Berk. & M.A. Curtis) Jülich	TU122511	1	2	1	L	DSP	CF	EM	EN	D1
	*Postia floriformis*	(Quél.) Jülich	TU120617	21	39	5	18E28M54L	95S5P	72C15Sn13F	46E41M13L	LC	
	*Postia fragilis*	(Fr.) Jülich	TU117295	73	>100	14	2E55M43L	59S41P	62C9Sn29F	31E61M8L	LC	
	*Postia guttulata*	(Peck) Jülich	TU111192	42	37	12	3E84M14L	49S51P	65C16Sn14F5L	37E26M37L	LC	
	*Postia hibernica*	(Berk. & Broome) Jülich	TU122822	14	5	0	M	SP	CF	EM	VU	D1
	*Postia leucomallella*	(Murrill) Jülich	TU129611	>100	>100	8	4E70M26L	12S87P	51Cn48F	26E65M9L	LC	
	*Postia ptychogaster*	(F. Ludw.) Vesterh.	TU118955	8	37	16	27E24M49L	75S22P3A	76C8Sn16F	30E62M8L	LC	
	*Postia rennyi*	(Berk. & Broome) Rajchenb.	TU121994	12	9	0	EML	SP	CSnF	M	LC	
	*Postia romellii*	M. Pieri & B. Rivoire	TU129819	29	20	0	EML	SP	CSnF	EML	DD	
	*Postia stiptica*	(Pers.) Jülich	TU122881	48	83	6	16E47M37L	86S7P1D4B2A	48C26Sn24F2L	56E39M5L	LC	
	*Postia tephroleuca*	(Fr.) Jülich	TU129589	72	>100	9	11E52M37L	67S15P1D12B5A	63C12Sn24F	24E68M8L	LC	
	*Postia undosa*	(Peck) Jülich	TU115562	14	61	4	8E61M31L	65S17P2D2B15A	73C3Sn23F	25E65M10L	LC	
	*Pycnoporellus alboluteus*	(Ellis & Everh.) Kotl. & Pouzar	TAAM197000	0	0	11		S	C		CR	D1
	*Pycnoporellus fulgens*	(Fr.) Donk	TU120127	>100	>100	>100	7E37M56L	85S3P1D10B2A	85C10Sn5F	24E56M18L	LC	
	*Pycnoporus cinnabarinus*	(Jacq.: Fr.) P. Karst.	TU118339	73	>100	19	96E4M	23D77B	20C2Sn78F	22E63M15L	LC	
	*Rhodonia placenta*	(Fr.) Niemelä, K.H. Larss. & Schigel	TU117293	17	31	42	19M81L	55S39P3D3A	90C3Sn6F	13E55M32L	LC	
	*Rigidoporus crocatus*	(Pat.) Ryvarden	TU117325	30	>100	34	4E58M39L	12S2P51D32B3A	93C7F	10E67M23L	LC	
	*Sarcoporia polyspora*	P. Karst.	TU117066	4	5	1	ML	SP	C	EML	EN	D1
	*Sidera lenis*	(P. Karst.) Miettinen	TU101553	31	6	4	ML	SP	CSnF	ML	VU	D1
	*Sidera vulgaris*	(Fr.) Miettinen	TU122882	69	79	20	3E51M47L	48S39P5D3B5A	62C38F	6E48M46L	LC	
^m*^	*Sistotrema alboluteum*	(Bourdot & Galzin) Bondartsev & Singer	TU121700	9	12	1	ML	SP	CF	EML	NT	D1
^m*^	*Sistotrema confluens*	Pers.	TU118544	31	0	20			G		LC	
^m*^	*Sistotrema dennisii*	Malençon	TAAM6601	1	0	0					DD	
^m*^	*Sistotrema muscicola*	(Pers.) S. Lundell	TAAM180781	2	6	1	ML	PD	CF	ML	NT	D1
	*Skeletocutis amorpha*	(Fr.) Kotl. & Pouzar	TU120616	>100	>100	16	18E64M18L	32S68P	60C24Sn16F	65E35M	LC	
	*Skeletocutis biguttulata*	(Romell) Niemelä	TU122884	60	>100	3	12E73M15L	7S89P2D2B1A	27C3Sn70F	26E55M19L	LC	
	*Skeletocutis brevispora*	Niemelä	TU117344	1	3	5	L	S	C	EML	CR	C2a(i)
	*Skeletocutis carneogrisea*	A. David	TU122445	65	>100	7	7E65M28L	69S31P	64C5Sn31F	23E74M3L	LC	
	*Skeletocutis cummata*	A. Korhonen & Miettinen	TU128007	4	1	0	M	S	SnF	M	EN	D1
	*Skeletocutis delicata*	Niemelä & Miettinen	TU129588	0	2	2	ML	SP	C	ML	NE	
	*Skeletocutis exilis*	Miettinen & Niemelä	TU129591	0	1	0	L	S	C	L	NE	
	*Skeletocutis jelicii*	Tortic & A. David	TU111188	0	1	3	M	S	C	L	EN	D1
	*Skeletocutis kuehneri*	A. David	TU129264	6	18	9	ML	SP	CF	EML	LC	
	***Skeletocutis nivea sl***			>100	>100	19	15E54M32L	75D19B5A	11C1Sn87F1L	34E52M14L	LC	
	*- Skeletocutis futilis*	Miettinen & A. Korhonen	TU129978									
	*- Skeletocutis nemoralis*	A. Korhonen & Miettinen	TU130512									
	*- Skeletocutis semipileata*	(Peck) Miettinen & A. Korhonen	TU122298									
	*Skeletocutis odora*	(Peck ex Sacc.) Ginns	TU117273	26	14	59	EML	SPA	CSn	EML	VU	A3c; C1
	*Skeletocutis papyracea*	A. David	TU122787	17	>100	6	2E79M19L	14S86P	54C1Sn45F	26E67M7L	LC	
	*Skeletocutis stellae*	(Pilát) Jean Keller	TU129605	43	16	33	EML	SP	CF	ML	NT	D1
^p^	*Spongipellis spumea*	(Sowerby) Pat.	TAAM189911	11	0	11		D	L		VU	D1
	*Steccherinum oreophilum*	Lindsey & Gilb.	TAAM158353	1	2	3	M	BA	F	M	DD	
	*Trametes gibbosa*	(Pers.) Fr.	TU117579	1	0	13		D			VU	D1
	*Trametes hirsuta*	(Wulfen) Pilát	TU120101	>100	>100	24	78E15M8L	1S37D48B13A	20C9Sn71F	49E44M7L	LC	
	*Trametes ochracea*	(Pers.) Gilb. & Ryvarden	TU120126	>100	>100	17	44E37M19L	1S8D50B42A	27C16Sn56F1L	56E40M4L	LC	
	*Trametes pubescens*	(Schumach.) Pilát	TU101930	58	69	3	55E38M7L	13D75B12A	36C9Sn53F2L	28E62M10L	LC	
^p?^	*Trametes suaveolens*	(L.) Fr.	TU128010	13	0	16		D	SnL		CR	CR C2ai; D1
	*Trametes versicolor*	(L.) Lloyd	TU118802	>100	>100	17	82E16M2L	2S43D53B2A	19C44Sn37F	28E47M25L	LC	
	*Trametopsis cervina*	(Schwein.) Tomsovský	TU109320	0	0	2		D			NE	
	*Trechispora candidissima*	(Schwein.) Bondartsev & Singer	TU123416	4	8	1	EML	SPDB	CSnF	EML	LC	
	*Trechispora hymenocystis*	(Berk. & Broome) K.H. Larss.	TU129229	56	82	3	22E52M26L	25S26P10D39B	36C10Sn53F	5E38M57L	LC	
	*Trechispora mollusca*	(Pers.) Liberta	TU129726	45	83	3	30E30M40L	44S9P9D36B3A	33C5Sn62F	11E51M38L	LC	
	*Trichaptum abietinum*	(Pers. ex J.F. Gmel.) Ryvarden	TU118911	>100	>100	30	15E57M28L	70S30P	50C19Sn32F	66E33M1L	LC	
	*Trichaptum biforme*	(Fr.) Ryvarden	TU120047	24	58	7	3E79M17L	3D97B	43C2Sn48F7L	30E55M15L	LC	
	*Trichaptum fuscoviolaceum*	(Ehrenb.) Ryvarden	TU118960	42	>100	12	26E69M5L	2S98P	31C21Sn48F	68E32M	LC	
	*Tyromyces chioneus*	(Fr.) P. Karst.	TU120543	83	>100	4	65E31M4L	1P10D81B8A	28C16Sn55F	14E58M28L	LC	
	*Tyromyces fumidiceps*	G.F. Atk.	TAAM189638	4	0	0					EN	D1
	*Xanthoporus syringae*	(Parmasto) Audet	TAAM159469	4	0	0					VU	C2a(i); D1

^1^ life history strategy: ^p^ parasite; ^m^ mycorrhizal; ^m*^partly mycorrhizal; ^?^ uncertain

We use conservative nomenclature for genera whose classification is still in flux, such as *Antrodia*, *Phellinus*, *Inonotus,* and *Polyporus*.

To update the species list, special attention was paid to specimens that represented taxa with recently updated taxonomy (notably the species concept) and potentially unresolved groups. Such specimens were checked microscopically, and multiple dried basiodiomes sequenced for rDNA ITS (in the case of high variability also D1–D2 domains of the more stable LSU region) for comparisons with references in public databases and our personal database. For obtaining the ITS sequences, we used primers ITS1F (Gardes & Bruns 1993) or ITS0F-T (Tedersoo et al. 2008) and ITS4 (White et al. 1990); for the D1–D2 domains of the LSU region we used primers CTB6 (Garbelotto et al. 1997) and LR7 (Vilgalys & Hester 1990) or LBW (Tedersoo et al. 2008). DNA extraction, polymerase chain reaction (PCR), and sequencing of the target loci followed protocols described by Tamm and Põldmaa (2013). ITS and LSU sequences were also produced for 82 species that had no previously sequenced voucher specimens from Estonia.

In eight difficult/unresolved species groups, we explicitly illustrate the variation in their Estonian ITS (in some cases also LSU) sequence material and the accompanying ecological data on substrate and habitat type. The sequences were edited and assembled using Sequencher 5.1 (Gene Codes, Michigan, USA), first aligned automatically using Mafft 7 online version (Katoh et al. 2017) and then edited manually in AliView (Larsson 2014). The Estonian dataset of each taxon group was complemented with the most similar basidiome based sequences (> 95% similarity) available at GenBank and UNITE database (Nilsson et al. 2018). In UNITE, a species hypothesis at 1.5% threshold level was calculated for a voucher specimen of each distinct lineage (Kõljalg et al. 2013). Outgroups were chosen based on the latest molecular taxonomic works on the target taxa, except in *Byssoporia*, *Coltricia cinnamomea*, *Physisporinus* and *Sidera* that had difficult to align ITS/LSU regions. To avoid rooting with distant taxa and producing arbitrary branching orders, their phylogenetic trees were centrally rooted. We organized the sequences as Maximum Likelihood (ML) phylogenies based on IQ-TREE (version 1.2.2; Nguyen et al. 2015), 1000 bootstrap replicates and the ‘best-fitted model’. Collection data for the examined Estonian specimens in difficult/unresolved species groups and the GenBank or UNITE accession numbers of their ITS and LSU sequences are presented in Additional file 3, data for public reference sequences from elsewhere are in Additional file 4. The final alignments for all data sets were stored in TreeBASE (http://www.treebase.org; accession number 25415).

##### Analysing polypore assemblages along habitat gradients

Primary data for assessing correspondence between polypore assemblages and habitat gradients were the systematic surveys in stands > 20 years old (datasets IIa-IIc in Table 1). We categorized the stands into ‘habitat types’ according to: (1) site-type group – proxy of soil nutrient and humidity combinations (Lõhmus 1984, Additional file 1); (2) tree canopy composition class – *Picea abies* forests and *Picea*-deciduous mixedwood; *Pinus sylvestris* forests and *Pinus*-deciduous mixedwood; deciduous forests (≥80% deciduous species); and (3) old stands (dominant tree layer > 100 years) vs. other stands. We then compiled species lists for each habitat type by pooling species data from all stands belonging to this type. Such approach allowed us to address relative importance of permanent (soil) and temporary variation (tree composition and successional stage) for polypore assemblages. We did not analyse the distinct post clear-cut assemblages that have been addressed in original studies (Lõhmus 2011, Runnel & Lõhmus 2017); the species found in such early-successional stands can be distinguished in Table 2.

Additionally, we compiled species lists for bog and heath forests, parks and wooded meadows, which have not been systematically surveyed. We used casual records extracted from PlutoF database and Parmasto (2004), relying on original habitat annotations (these habitat types are easily distinguishable); we nevertheless double-checked all such records that had co-ordinates against the Estonian soil map. Tree composition and age were not specified for these additional data, but heath and bog forests in Estonia are typically *Pinus sylvestris* stands, while most parks and wooded meadows characteristically have old deciduous trees.

Overlaps of species lists among site-type groups were visualized with Euler proportional circle diagrams (eulerr package; Larsson 2018). For assemblage analyses along habitat gradients, we first omitted all species that had been recorded from a single habitat type (a combination of 1–3 above). This retained data on 157 polypore species with 23,362 original records and 54 habitat types. We then recoded species’ record numbers for a three-class scale (0, no records; 1, one record; 2, > 1 records) as a compromise between observation bias in raw record numbers (resulting from varying habitat coverage and species detectability) and the presence-absence scale’s emphasis on rare species.

To illustrate how assemblage composition varies among habitat types, we used non-metric multidimensional scaling (NMDS; vegan package in R, Oksanen et al. 2016). The environmental matrix comprised three categorical variables: site-type group (ten groups; Additional file 1), soil fertility (two classes: fertile vs poor/thin), and tree species composition (three classes, see above). The analyses were run using the Bray-Curtis dissimilarity index with random starting configurations; searching for two-dimensional solutions, and rotating the final solution to depict the largest variance of site scores on the first axis. Assemblage differences were tested separately for each environmental variable using Multi-Response Permutation Procedures (MRPP) with Bray-Curtis dissimilarity index, and Bonferroni corrected *p*-values.

#### Substrate analyses

We followed the concept of functional traits as presented by Dawson et al. (2019) and categorized species mostly according to Niemelä (2016). We first divided the species between strictly or facultatively ectomycorrhizal and wood-inhabiting life-strategy groups. The wood-inhabiting group was further divided by: (a) typical colonization time – parasites of live trees (‘necrotrophs’ sensu Dawson et al. 2019), early-decayer (most records on trees of decay stage I–II) and late-decayer saprotrophs (stage III–V); and (b) physical decay strategy – white-rot and brown-rot producing species. The saprotrophs include some polypores that are frequent on very fine debris, and some ‘follower’ species that require wood decayed by other parasitic or saproxylic basidiomycetes (Holmer et al. 1997, Niemelä 2016).

We pooled all the available polypore records on naturally developed woody substrates, excluding building timber for which we only report the state of the knowledge. The records are from the datasets I-IIIa (Table 1) and, for *Juniperus communis*, as summarized by Sell & Koti-ranta (2011). Host tree species have been indicated in all these datasets. We additionally distinguished the main woody fractions and decay stages – those data mostly originate from the systematic surveys (datasets IIa-d). We re-coded the decay stages I–II sensu Renvall (1995) as ‘early’, III as ‘medium’, and IV–V as ‘late’; in the latter we also included casual records describing the wood as “extremely decayed”. Fine woody debris (FWD) includes both fallen and standing dead wood items < 10 cm in diameter at the basidiome location.

Based on the distribution of records among all substrate categories, we distinguished regularly occurring and specialist polypores for a substrate category as follows. ‘Regular’ species, either: had ≥5% records on that substrate category of the species’ total of ≥40 records in Estonia, or had > 1 records there of its total of < 40 records, or accounted for ≥5% of all polypore records in that substrate category. ‘Specialists’ were a subset of regular species, which had > 2 records from a particular substrate category and this formed either ≥90% of all Estonian records of that species, or all records if the total number of records was 3–9.

Similarity of polypore species composition of native host tree species was further explored with hierarchical cluster analysis based on presence-absence data, Bray-Curtis dissimilarity measure and the average linkage method (r package vegan; Oksanen et al. 2016). Because presence-absence data would over-emphasize atypical substrates, only polypores occurring regularly on each tree species (≥5% of total records in the tree or polypore species) were included in this analysis.

## RESULTS

### Estonian polypore diversity

Parmasto (2004) reported 212 polypore species in Estonia, of which 198 can be currently considered accepted, although several have been subdivided on a larger geographical scale (e.g. *Antrodia crassa, Antrodia sitchensis*, *Polyporus tuberaster, Postia sericeomollis* and *Skeletocutis nivea s. str.* are not known in Estonia). Six of those species are now listed as Regionally Extinct based on the lack of records for > 50 years: *Antrodia heteromorpha*, *Diplomitoporus crustulinus*, *Inonotopsis subiculosa*, *Inonotus dryadeus, Phellinus viticola*, and *Polyporus pseudobetulinus* (Table 2). Probably, they were already extinct in 2004. Excluded species include seven formerly recognized taxa (*Antrodia albida, Ceriporia subreticulata, Phellinus cinereus*, *Postia lactea*, *Sistotrema albopallescens, Skeletocutis subincarnata*, and *Trametes velutina*) that are now merged with other species known in Estonia. We also excluded two putative new *Phellinus* species on Parmasto’s list (status as independent species not supported). Five species were excluded because the historical material had been misidentified: *Antrodiella canadensis, Ganoderma adspersum,* and *Skeletocutis alutacea* (all specimens checked), and *Postia lateritia* and *Trichaptum laricinum* (most specimens checked, none confirmed)*.* Two species, *Aurantiporus priscus* (a part of “*Hapalopilus salmonicolor*” records in Parmasto 2004) and *Ganoderma carnosum*, remain on our list based on Parmasto’s original identifications; the collections have survived but we failed to obtain sequences from this old material.

As of July 2019, the list comprises 221 verified extant species (Table 2), including 11 with no post-2004 records (*Anomoloma albolutescens, Antrodiella parasitica*, *Aurantiporus priscus, Ganoderma carnosum*, *Oxyporus ravidus*, *Perenniporia tenuis, Polyporus submelanopus*, *P. tubaeformis, Sistotrema dennisii, Tyromyces fumidiceps,* and *Xanthoporus syringae*). Seventeen extant species have been only recorded once, and six only twice (Table 2); 11 of these extremely rare species were recorded in 2005–18. Based on the numbers of accepted species, singletons and doubletons, the Chao (1987) estimate for expected species richness is 245 extant species. Additionally, there are records of at least 20 lineages that may deserve species status (see under Difficult species below; Table 3). Three species are, according to current records, restricted to the West-Estonian, and nine to the East-Estonian geobotanic regions (only species with > 1 records considered).

**Table 3:** Lineages of unnamed and/or collective polypore species in Estonia. Freq – no. of records in Estonia (* 1; ** 2–5; *** > 5)

	Vouchers from Estonia				Best match from outside Estonia			
Taxon	Lineage	Voucher ID	UNITE SH code at 1.5% threshold level	Similarity % (no. of variable/total sites)	Annotation	GenBank no.	Similarity to voucher % (no. of variable/total sites)	Freq.
*Antrodiella faginea*	L1	TU130324	SH1600328.08FU	99% (6/549 BP)	*A. faginea* (CZ)	AF126885	100% (0/549 BP)	***
	L2	TU130481	SH1600328.08FU		*A. faginea* (RU)	KU726586	100% (0/547 BP)	***
*Byssoporia terrestris*	L1	TU130505	SH1542891.08FU	79% (124/583 BP)	*B. terrestris* (FI)	UDB031621	99% (2/576 BP)	*
	L2	TU130449	SH1629432.08FU		*B. terrestris* (SE)	EU118608	83% (101/587 BP)	**
*Ceriporia excelsa*	L1 (s. typi)	TU115577	SH2141340.08FU	98% (15/909 BP)	*C. excelsa* (US)	MH858306	100% (0/598 BP)	***
	L2	TU124431	SH1510726.08FU		*Ceriporia* sp. (US)	KP135050	99% (2/598 BP)	*
*Ceriporia viridans*	L1 (s. typi)	TU130515	SH1510720.08FU	97% (27/902 BP)	*C. viridans s str.* (FI)	KX236481	99% (4/549 BP)	***
	L2	TU130057	SH1510723.08FU		*C. viridans* s str. (FI)	KX236481	97% (23/549 BP)	***
*Ceriporiopsis pseudogilvescens*	L1 (s. typi)	TU122449	SH1543621.08FU	99% (2/597 BP)	*C. pseudo-gilvescens* (CN)	KU509523	100% (0/597 BP)	***
	L2	TU129148	SH1543621.08FU		*C. resinascens* (SK)	FJ496679	99% (2/597)	**
*Coltricia cinnamomea*	L1	TU110786	SH1651067.08FU	76–99% (8–151/574 BP)	*C. cinnamomea* (CN)	KY693732	88% (72/584 BP)	**
	L2	TU113488	SH1651067.08FU		*C. cinnamomea* (CN)	KY693732	87% (73/580 BP)	**
	L3	TU106861	SH1611633.08FU		*Coltricia* sp. (MX)	MG966155	98% (12/595 BP)	*
	L4	TAAM196949	SH1651068.08FU		*C. cinnamomea* (CN)	KY693729	90% (63/608 BP)	*
*Coltricia perennis*	L1	TU106858	SH1554196.08FU	86–99% (8–76/553 BP)	*C. perennis* (US?)	DQ234560	99% (8/541 BP)	*
	L2	TU110835	SH1554196.08FU		*C. perennis* (US?)	DQ234560	100% (0/538 BP)	*
	L3	TU106860	SH1554198.08FU		*C. perennis* (FI)	MF319057	99% (2/543 BP)	**
*Physisporinus sanguinolentus*	L1	TU122889	SH1558568.08FU	97% (19/543 BP)	*P. furcatus* (RU)	KY131853	98% (12/532 BP)	**
	L2	TU129782			*P. furcatus* (CN)	KY131856	99% (5/536 BP)	*
*Physisporinus vitreus*	L1	TU130068	SH1615294.08FU	94–99% (2–27/464 BP)	*P. sanguinolentus* (SE)	JX109843	99% (5/541 BP)	***
	L2	TU129958	SH1615294.08FU		*P. sanguinolentus* (SE)	JX109843	99% (4/541 BP)	**
	L3	TU130572	SH1615294.08FU		*P. sanguinolentus* (SE)	JX109843	99% (1/463 BP)	**
	L4	TU122877	SH1615296.08FU		*P. sanguinolentus* (SK)	FJ496671	100% (1/539 BP)	**
*Sidera* spp.	L1 (annual)	TU122801	SH1544622.08FU	80–86%% (90–176/871 BP)	*Sidera* sp. (US)	KP814157	97% (15/597 BP)	**
	L2 (annual)	TU129576	SH1612214.08FU		*Schizopora* sp. (US)	MF161274	99% (4/587 BP)	***
	L3 (perennial)	TU122545	SH1540362.08FU		*Sidera vulgaris* (AU)	FN907922	96% (12/280 BP)	***
*Sistotrema alboluteum*	L1	TU121700	SH1506830.08FU	95% (28/538 BP)	*S. aff. alboluteum* (US)	KP814533	94% (30/538 BP)	**
	L2	TU130503	SH1506832.08FU		*S. aff. alboluteum* (US)	KP814533	99% (2/534 BP)	*
*Sistotrema muscicola*	L1	TU130567	SH1539308.08FU		*Sistotrema* sp. (US)	KP814242	91% (48/533 BP)	**
	L2	TAAM180781	SH1506835.08FU	85–94% (30–72/530 BP)	*S. muscicola* (FI)	AJ606040	99% (1/475 BP)	*
	L3	TAAM202939	SH1539286.08FU		*Sistotrema* sp. (US)	KP814241	91% (51/537 BP)	**
	L4	TU130466	SH1539297.08FU		*Sistotrema* sp. (US)	KP814241	91% (47/525 BP)	*
*Skeletocutis* sp. (*kuehneri* group)		TU128024	SH1541633.08FU		*Skeletocutis chrysella* (FI)	FN907916	95% (28/583 BP)	*

Species were added to the 2004 list for three reasons (* solely from casual collections) and include two species newly reported for Europe (*Polyporus submelanopus*, *P. ulleungus*):

1. Ten established species were found in nature for the first time after 2004: Coltricia cinnamomea, C. confluens, Fibroporia norrlandica, Obba rivulosa, Perenniporia narymica, Phellinus hippophaeicola*, Postia auricoma, Pycnoporellus alboluteus*, *Skeletocutis jelicii,* and Trametopsis cervina*. Eight of these (excluding *F. norrlandica* and *O. rivulosa*) are easy to find and identify, and may thus constitute true recent additions to the Estonian mycota.
2. Nine species have been distinguished from other species present in the area and confirmed or likely to be present in the pre-2004 material of the collective species: *Ceriporia bresadolae* (from *C. purpurea*), *Hapalopilus aurantiacus* and *H. ochracolateritius* (from “*H. salmonicolor*” sensu Parmasto 2004), *Postia cyanescens*, *P. simulans* and *P. populi* (from *P. alni* and *P. caesia*), and *Skeletocutis futilis*, *S. nemoralis* and *S. semipileata* (from *S. nivea s. str.* that is not known in North Europe).
3. Ten species, now confirmed in Estonia, have been described or reinstated only after 2004. Of these, *Antrodia leucaena* has been confirmed by us also in the Estonian pre-2004 material, and Polyporus submelanopus* only in that material. The other species are: *Aporpium macroporum*, *Ceriporia aurantiocarnescens, C. torpida, Junghuhnia autumnale, Leptoporus erubescens, Polyporus ulleungus, Skeletocutis delicata,* and *S. exilis*.

### Difficult species

We distinguished 13 species groups of Estonian polypores, for which the assessment of population status and ecology was complicated (details in Additional file 5). In most cases, the problem was unresolved taxonomy: molecular data revealed that the prevailing species concept included cryptic lineages (Table 3, Additional file 3), some with documented ecological differences. Specifically, Estonian specimens referred to in Table 2 by the accepted names *Antrodiella faginea*, *Byssoporia terrestris*, *Ceriporia excelsa*, *C. viridans*, *Ceriporiopsis pseudogilvescens, Physisporinus sanguinolentus*, *Sidera vulgaris*, and *Sistotrema alboluteum* represented two distinct lineages each, and those identified as *Coltricia cinnamomea*, *C. perennis*, *Physisporinus vitreus* and *Sistotrema muscicola* at least three lineages each. Additionally, we sequenced an undescribed lineage related to *Skeletocutis kuehneri*/*brevispora*, and found that the Estonian specimens of *Sidera lenis* do not match with its prevailing species concept. In the *Ceriporiopsis resinascens* / *C. pseudogilvescens* lineages, the main morphological characteristics represented a continuum and some specimens had ITS copies from multiple lineages. The abundance of records or their habitat diversity indicated no apparent conservation concern in any lineages of *Antrodiella faginea* and *Ceriporia viridans*, while at least one likely threatened lineage was detected in *Ceriporia excelsa*, *Coltricia cinnamomea*, *C. perennis*, and *Sidera vulgaris.*

Another, sometimes combined problem was the lack of stable morphological character combinations to enable species identification in recently revised species groups; this introduced large uncertainty to interpreting historical collections and observations. For example, the species earlier known as *Postia caesia, P. alni, P. leucomallella*, and *Skeletocutis nivea* have been considered easily identifiable in the field and their mostly observational data cannot be ascribed to the recently segregated species. Also, sequencing of European fungarium specimens of black-stiped *Polyporus* collections is recommended due to high likelihood of finding species traditionally not considered to occur in Europe.

### Functional traits

Most Estonian polypore species produce annual basidiomes, but in 51 species these survive for at least 2–3 years (usually > 3 years in 33 of these). The prevailing life strategy is saprotrophy, with at least 12 species being follower species of other wood-inhabiting (parasitic or saprotrophic) polypores (Table 2). Based on systematic surveys (datasets IIa–c; Table 1), basidiomes of the follower species are found 1–3 orders of magnitude less frequently than their predecessor species. A wide variation can occur in the same predecessor species, e.g., the Estonian records among the followers of *Trichaptum abietinum* range from one (*Antrodiella parasitica*) to 380 (*Skeletocutis carneogrisea*).

Thirty-four polypore species are parasites of live trees or shrubs, but usually continue living as saprotrophs after death of the host-tree. Three parasitic species (*Heterobasidion annosum*, *H. parviporum,* and *Phellinus tremulae*) are considered economically important forest pathogens in Estonia. Thirteen polypore species are considered strictly or facultatively ectomycorrhizal (*Albatrellus*, *Boletopsis*, *Coltricia,* and *Sistotrema*) (Table 2). Distinctly among functional groups, mycorrhizal polypores are most diverse in dry and low-productive forest types: eight species inhabit alvar forests (on calcareous soil), eight dry boreal, and seven boreal heath forests (on sandy soil). In contrast, only three mycorrhizal species have been found in eutrophic sites, five in meso-eutrophic, and three in swamp forests.

### Habitat types and assemblages

Among the three broad forest successional stages (Table 2), the largest numbers of species have been recorded in mid-successional forests (146; incl. 16 parasitic and five wholly or partially mycorrhizal species) and late-successional forests (146; incl. 19 parasitic and eight wholly or partially mycorrhizal species). The largest numbers of threatened species were found in late-successional (38 species) and mid-successional forests (34). Based on systematic surveys (Table 1: datasets IIa–c), the most abundant species in mid- and late-successional forests are *Fomitopsis pinicola* (10.5% of 18,026 records), *Trichaptum abietinum* (8.5%), and *Fomes fomentarius* (7.4%). In post clear-cut (early-successional) stands, most abundant are *Gloeophyllum sepiarium* (9.0% of 4939 records), *F. pinicola* (7.5%), and *Trametes hirsuta* (6.4%). However, these proportions are underestimates compared with rarer species, since our sampling included up to ten records of each species per plot (see Methods).

**Fig. 2:**
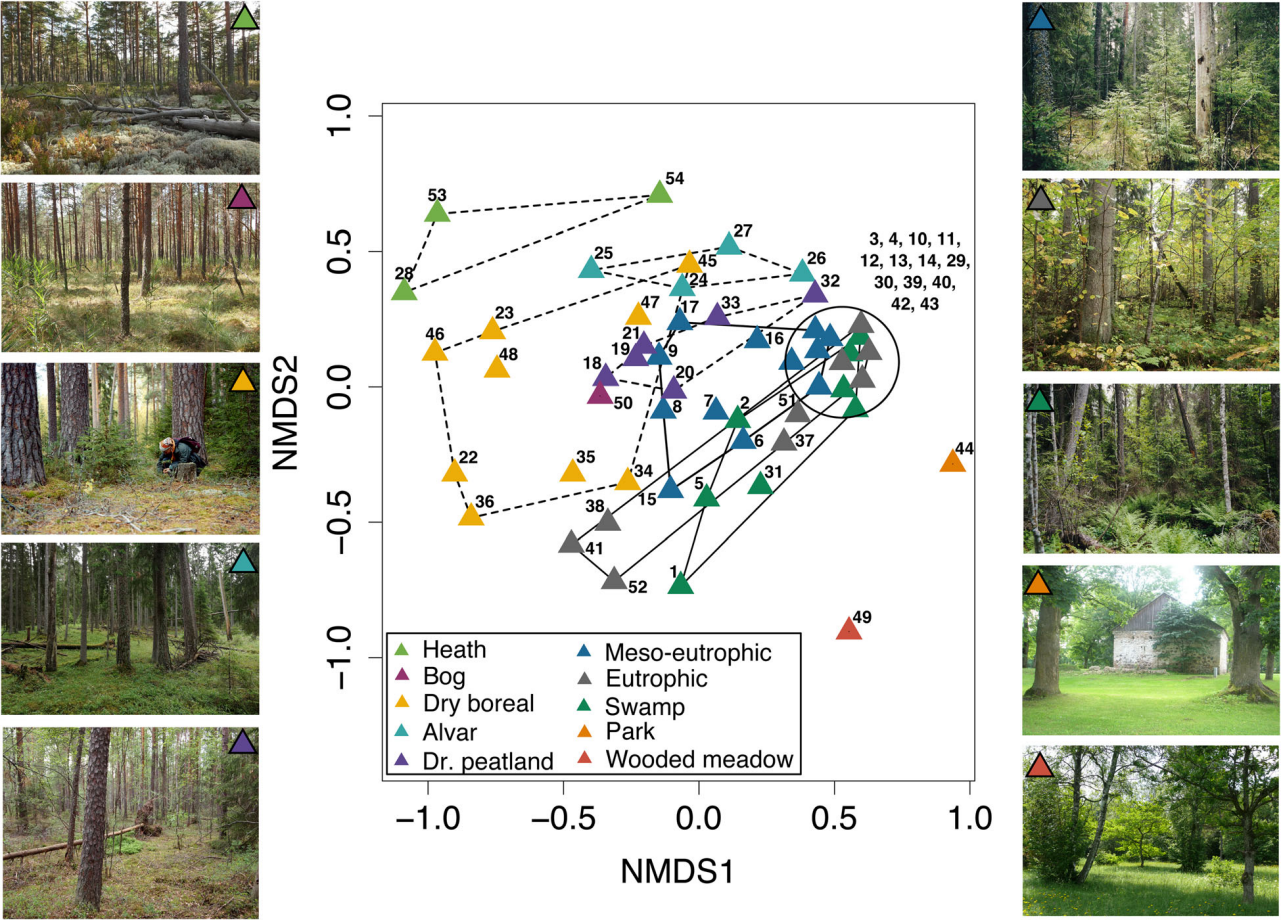
Non-metric multidimensional scaling (NMDS) ordination diagram of polypore assemblages in 54 site-type group × tree species × age combinations (points; the number codes explained in Additional file 1). The two-dimensional solution with the final stress value of 0.166 is shown. The symbols denote woodland types; photo credits: E. Lõhmus, P. Lõhmus, A. Palo. Note the three woodland types represented by a single pooled species list: parks (44), wooded meadows (49) and bog forests (50)

Estonian polypore assemblages in > 20 year-old forests are primarily organized along the soil (site type) and tree species composition gradients (Fig. 2; Additional file 6). The first ordination axis broadly distinguished assemblages on fertile soils from those on poor soils (sandy, thin calcareous, or peat soils) (MRPP test: A = 0.08, *p* < 0.001). The second axis ranged from deciduous- to *Pinus*-dominated stands, with *Picea*-dominated forests in the middle (MRPP tests: A = 0.07–0.09, p < 0.001, for the contrasts with *Pinus*-dominated sites; A = 0.03, *p* = 0.02, for *Picea*- vs deciduous-dominated sites). These two gradients overshadowed soil moisture effects; e.g., *Pinus*-dominated sites with contrasting moisture conditions (dry alvar forests, wet drained peatland, and bog forests) were positioned close to each other, but clearly apart from moist sites dominated by either *Picea* or deciduous trees (Fig. 2).

**Fig. 3:**
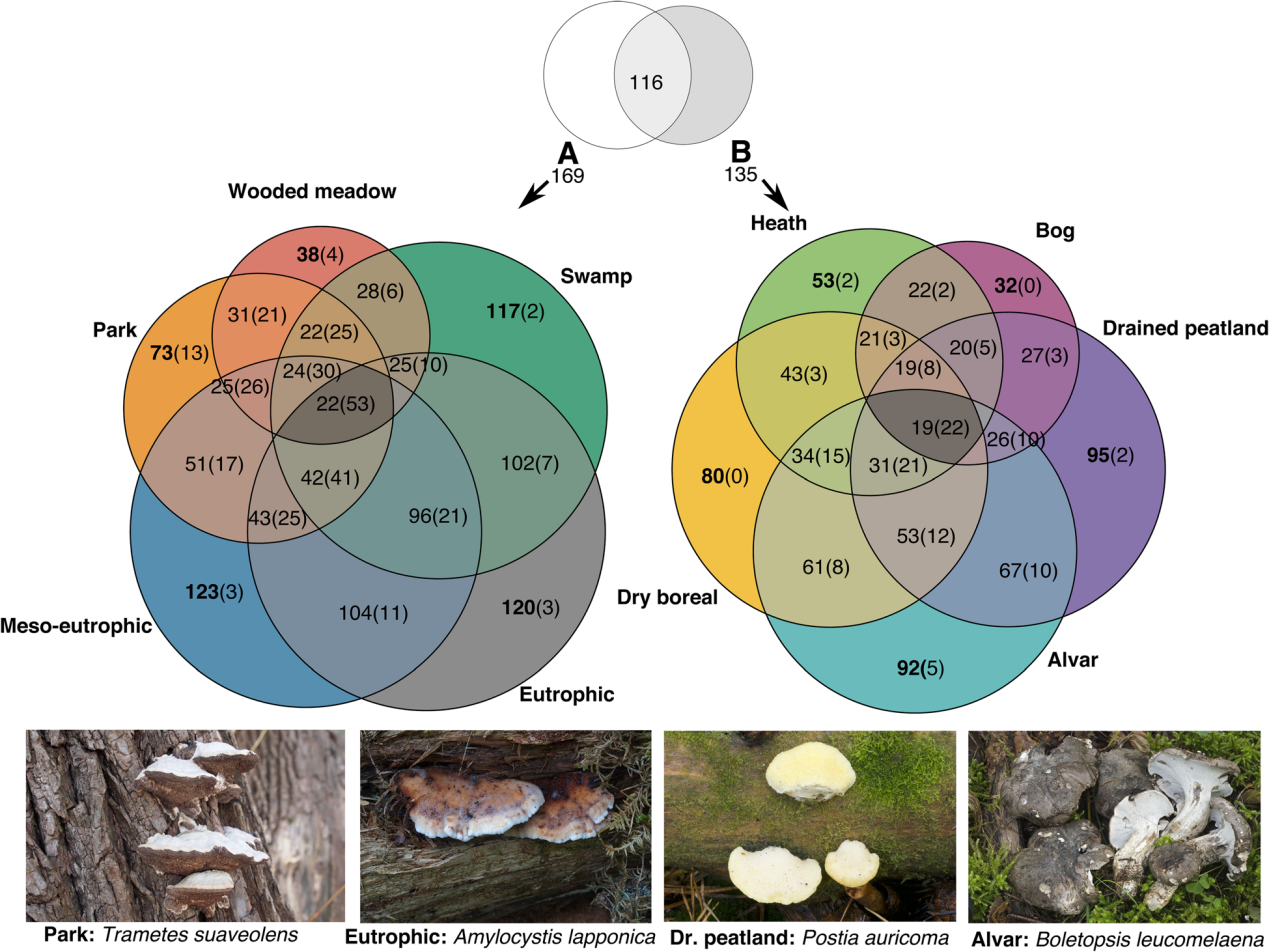
Euler diagrams of 189 polypore species (including singletons) found in different combinations of Estonian habitat types on fertile soils (**a**) and poor soils (**b**). The numbers before parentheses indicate species found in every habitat type included in the combination; the numbers in parentheses indicate species that have not been found elsewhere (considering both types of soils); examples are illustrated on the photos (Photo credit: V. Liiv, E. Lõhmus, O. Miettinen, U. Ojango). The habitat combinations shown were extracted by the eulerr package (Larsson 2018); see Additional file 7 for statistics of other habitat combinations

Across natural forest types, polypore assemblages formed a continuum in the ordination space (Fig. 2), i.e., only distant types differed significantly from each other. For example, the assemblages in eutrophic sites appeared close to those in meso-eutrophic or swamp sites (MRPP: A ≤ 0.01, Bonferroni corrected *p* > 0.1), but differed from all other forest site-type groups (A = 0.09–0.14, *p* < 0.033 in all comparisons). Such a pattern is also revealed on the Euler diagrams: increasing proportions of species common to more similar site types, but a relatively small number of generalists across all habitat types (Fig. 3A,B middle section). The most distinct assemblages in natural forests were in alvar forests that differed from all others (A = 0.08–0.17, *p* < 0.034 in all comparisons), except perhaps heath forests (A = 0.13, *p* = 0.067). Specific species in our sample of alvar forests were the ectomycorrhizal *Albatrellus citrinus* and *Boletopsis leucomelaena*, and saprotrophic *Anomoloma myceliosum* and *Skeletocutis jelicii*. The largest number of habitat-specific species inhabit natural forests on nutrient-rich soils: 21 such species in eutrophic and swamp sites combined, including 18 extremely rare or threatened species (e.g. *Picea*-inhabiting *Amylocystis lapponica*, *Antrodia piceata,* and *Skeletocutis brevispora*; *Populus*-inhabiting *Aporpium macroporum*, *Junghuhnia fimbriatella,* and *Inonotus rheades*).

All anthropogenic woodland types (drained peatland forests, parks, and wooded meadows) hosted distinct polypore assemblages (Fig. 2). Drained peatland forests revealed two specific species (*Postia auricoma*; *Antrodia macra*) and their full assemblages resembled most those in dry boreal (MRPP test: A = 0.04, *p* = 0.060) or meso-eutrophic forests (A = 0.04, *p* = 0.069), while all other forest site-type groups were dissimilar (A = 0.09–0.11, *p* < 0.035). Parks and wooded meadows were each represented with one pooled species list in our data; thus we could not formally test their assemblage differences. However, as illustrated by the Euler diagrams (Fig. 3), parks had the largest number of specific species (13) and seven polypores are largely confined to large oaks (*Quercus robur*) and elms (*Ulmus glabra*) typical of parks and wooded meadows (*Daedalea quercina*, *Fistulina hepatica*, *Grifola frondosa*, *Inonotus ulmicola*, *Phellinus robustus, Perenniporia medulla-panis*, and *Polyporus umbellatus*). Some of the latter species also inhabit the rare natural oak stands in Estonia, which have not been systematically surveyed; casual data show that such stands additionally host some highly threatened species (*Aurantiporus croceus* and *Haploporus tuberculosus*).

### Woody substrates and substrate specificity

Host tree species data were available for 204 Estonian polypore species that inhabit natural woody substrates (Table 4). Sixty (29%) of these species can be considered tree-species specialists*. Picea abies* stands out with most associated species (108) and threatened species (40), and one-third of all specialist species (20, including 11 threatened species). The other polypore-rich trees include *Pinus sylvestris* and *Betula* spp. (the most abundant tree species in Estonia) and *Populus tremula*. *Quercus robur* is the only other tree species with several specialist polypores recorded. In contrast, small-sized woody species – shrubs and trees, which mostly stay in forest understories – generally lack specialist polypores (*Botryodontia millavensis* on *Juniperus communis* being the only exception). *Phellinus tuberculosus* and *Phylloporia ribis* are two specialized polypores so far only reliably recorded on fruit trees and shrubs in gardens (Table 4), although both have potential congenerous wild hosts in woodlands (*Prunus padus/spinosa* and *Ribes* spp., respectively).

**Table 4:** Numbers of polypore species recorded on naturally developed woody substrates in Estonia. The most species rich substrate in each substrate category (column) is indicated with bold script. Species counts by substrate type and decay stage may not correspond to the pooled species count of a tree species since some records lacked detailed substrate data. See Methods for the criteria of ‘regular’ and ‘specialist’ species. ‘-‘no information

		No. of species (no. of red-listed species: NT-RE)									
		Substrate types pooled			Substrate type				Decay stage		
		All	Regular	Specialist	Fallen trunk	Snag, stump	FWD	Live tree	Early	Medium	Late
Native woody species	*Picea abies*	**108(40)**	**73(26)**	**20(11)**	**101(38)**	54**(11)**	57**(13)**	13(1)	**66(17)**	**79(21)**	61**(17)**
	*Populus tremula*	102(23)	58(13)	11(8)	85(21)	44(6)	59(10)	**17**(3)	56(3)	67(10)	42(11)
	*Betula* spp.	97(18)	67(8)	8(0)	77(12)	**58**(3)	**68**(5)	16(1)	62(4)	75(8)	**69**(7)
	*Pinus sylvestris*	89(25)	60(16)	10(1)	77(23)	35(3)	54(11)	8(2)	56(10)	61(15)	40(8)
	*Alnus glutinosa*	71(7)	46(4)	0	60(6)	37(3)	48(3)	9(0)	39(5)	47(4)	40(2)
	*Alnus incana*	54(5)	19(0)	0	35(3)	20(0)	32(1)	2(0)	22(1)	23(0)	13(0)
	*Quercus robur*	54(14)	16(5)	6(3)	27(5)	17(2)	17(3)	12**(6)**	5(0)	3(0)	0
	*Salix* spp.	52(7)	14(1)	1(0)	30(3)	16(2)	32(3)	9(1)	14(2)	21(2)	7(1)
	*Fraxinus excelsior*	50(6)	18(2)	0	33(4)	20(1)	25(2)	7(0)	22(1)	27(2)	9(3)
	*Corylus avellana*	49(3)	17(2)	0	22(1)	16(1)	38(3)	5(0)	24(0)	22(2)	15(1)
	*Tilia cordata*	41(1)	10(1)	0	24(1)	16(1)	25(0)	7(0)	21(0)	23(1)	14(1)
	*Sorbus aucuparia*	40(2)	7(0)	0	18(1)	10(0)	23(1)	4(0)	18(2)	19(0)	8(0)
	*Acer platanoides*	33(4)	10(1)	0	21(2)	14(1)	9(0)	5(1)	9(0)	11(1)	5(0)
	*Ulmus* spp.	30(5)	7(2)	1(1)	14(2)	11(1)	6(0)	5(3)	5(0)	7(1)	1(0)
	*Prunus padus*	19(0)	3(0)	0	9(0)	6(0)	4(0)	2(0)	1(0)	3(0)	1(0)
	*Juniperus communis*	16(5)	5(1)	1(1)	4(1)	2(0)	8(2)	1(0)	7(3)	2(1)	2(1)
	*Frangula alnus*	7(0)	1(0)	0	0	2(0)	5(0)	0	6(0)	1(0)	1(0)
Exotic woody species	Deciduous	31(6)	3(1)	0	7(1)	7(0)	3(0)	10(2)	–	–	–
	Fruit trees, bushes	20(2)	3(0)	2(0)	6(0)	11(0)	0	12(2)	–	–	–
	Coniferous	14(2)	1(1)	0	5(1)	3(0)	0	2(1)	–	–	–
	TOTAL	204 (78)*	175 (57)	60 (25)	186 (67)	134 (30)	145 (35)	70 (16)	139 (33)	153 (39)	127 (30)

*In addition three species are known from unidentified tree species only

**Fig. 4:**
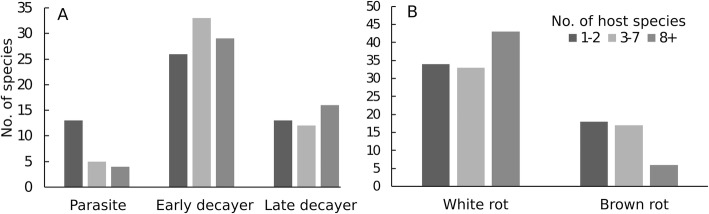
No. of host tree species listed for wood-inhabiting polypore species with > 10 records in Estonia by life strategy (**a**) and by decay type (**b**). The categorization for each species given in Additional file 8

Among 152 wood-inhabiting species recorded > 10 times in Estonia, 52 (34%) have been found on 1–2 tree species, 50 (33%) on 3–7 tree species, and 49 (32%) on at least 8 tree species. *Bjerkandera adusta* (recorded on 18 host tree species), *Trametes hirsuta* (18), and *T. versicolor* (16) had the widest host range. Host-tree specificity differs among functional groups: parasitic polypores are most often restricted to 1–2 tree species (Fig. 4a), and white-rot producers are more often generalists than brown-rot producers (Fig. 4b).

**Fig. 5:**
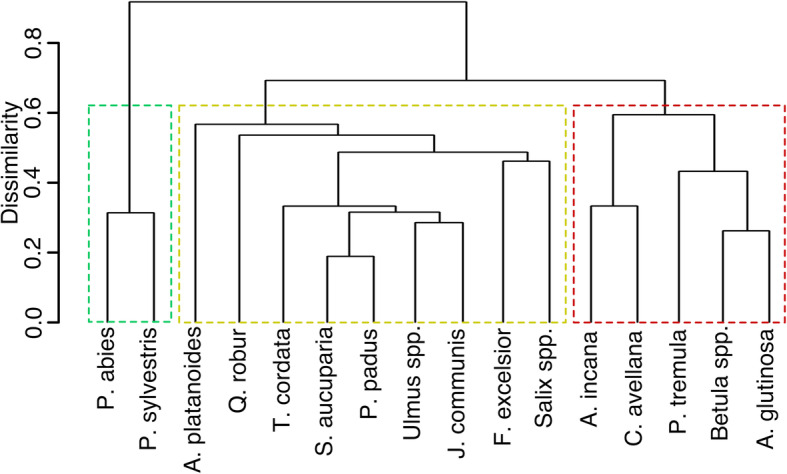
Similarity of polypore species composition on Estonian native tree species according to cluster analysis (average linkage method; Bray-Curtis dissimilarity). The main clusters of conifers, common soft-wooded deciduous tree species, and remaining tree or shrub species are indicated by coloured rectangles

By their polypore assemblages, native woody hosts form three main clusters that largely follow taxonomic divisions (Fig. 5): (1) the two Estonian conifer trees of *Pinaceae*; (2) common soft-wooded deciduous trees, including all native trees of *Betulaceae* (*Betula* spp. and *Alnus glutinosa* being the most similar host pair) and *Populus tremula* (a distinct host); and (3) the remaining woody species, with the most distinct assemblages on nemoral hardwoods (*Acer, Quercus*, and *Fraxinus*); *Salix* spp. clustering together with *Fraxinus*; and a similar host pair of the native trees in *Rosaceae* – *Prunus padus* and *Sorbus aucuparia*.

Coarse downed wood (fallen trunks) is by far the most polypore-rich woody fraction, with the largest number of species found in the medium decay stage (Table 4). This is despite a wider range of host species (including shrubs) providing fine woody debris. *Betula* spp. differs from other main tree species by distribution of species richness among wood fractions: relatively many species on fine woody debris and in late decay stages. Parasitic polypores are relatively diverse on *Quercus* and exotic (ornamental) deciduous trees, but the scarcity of records among wood decay stages in these trees mainly shows poor substrate documentation.

In addition to natural substrates, there are observations of polypores on building timber. From 2002 to 2008, Pilt et al. (2009) reported four species as regular in wooded buildings: *Antrodia serialis, A. sinuosa*, *Fibroporia vaillantii*, and *Fomitopsis pinicola*. Parmasto (2004) additionally mentions rare occurrences of *Fibroporia gossypium* and *Trametes ochracea* as well as “*Ceriporia purpurea”* (probably *C. bresadolae*) on building timber in Estonia.

## DISCUSSION

Our review demonstrates how integrating multiple data sources and their taxonomic and ecological appraisal can provide new perspectives on fungal species pools and their long-term dynamics. The practical opportunities discussed below included: posing new taxonomic and ecological hypotheses; fixing a state in the fungal biota for biodiversity monitoring purposes and retrospectives; providing a basis for red-listing individual species that considers all available data. The conservation issues can be further elaborated for management, which has been addressed elsewhere (Lõhmus et al. 2018b). Assessing the main factors behind changes in species lists helped us to understand actual changes in the biota and to prioritize research. We conclude that the Estonian polypore biota comprises over 260 species, of which roughly two-thirds were known 15 years ago according to their current species concepts, while the remaining third is divided between newly collected species, species distinguished from formerly known taxa, molecularly documented but yet-undescribed lineages, and species probably present but remaining to be found. Adding environmental DNA-samples to our basidiome data could be a next step to clarify the situation (cf. Kalsoom Khan et al. 2020).

### Estonian polypore biota as a part of the north-European species pool

The composition of the current Estonian polypore biota can be primarily explained through their woodland habitats and fungal biogeography. Both these patterns refer to post-glacial vegetation development, notably the climate- and land use-driven transformation of Estonian forests during the last millennium (e.g., Reitalu et al. 2013). It remains poorly known how fungal distributions have responded to this history, but some insight can be obtained based on comparisons of current regional biotas.

We documented 221 polypore species and > 20 to be described in Estonia. Comparing ours with the checklists in the neighbouring countries reveals extensive overlap of polypore biota across North-Europe, but clear latitudinal and longitudinal variation in relative abundance of species. Both the Finnish and Norwegian list include 251 species (Niemelä 2016, Tom Hofton, pers. comm.); but at least in Finland fewer species with 1–2 records than in Estonia (calculated from Niemelä 2016). Nevertheless, all the country lists now appear rather complete and the total species pool in Norway, Sweden (excluding its nemoral southern part), Finland and Estonia might be around 300 currently accepted species.

The part of this North-European species pool not found in Estonia comprises: (1) ca. 20 species having northern or north-eastern distributions in boreal forests; (2) several species having southwestern distribution in the Baltic Sea region, with Fennoscandia records mostly in southern Sweden; and (3) many extremely rare species having poorly explained scattered occurrences in Fennoscandia. Assigning the six species now considered Regionally Extinct in Estonia to the same groups reveals a disproportionate loss of northern species, with only *Inonotus dryadeus* representing group (2). Latitudinal patterns are further reflected by several southern species found in Estonia, but not in south Finland less than 100 km north. Of such species, *Abortiporus biennis, Coltricia confluens*, *Haploporus tuberculosus,* and *Perenniporia narymica* are also present in south Sweden (cf. group 2 above), and only *Oxyporus latemarginatus* and *Trametopsis cervina* have no Fennoscandian records at all. Some of these species are thermophilous; e.g., *Gloeophyllum trabeum* is confined to warm wooden indoor facilities in Finland but has a viable sexually reproducing population in the Estonian nature.

Longitudinal patterns are less apparent and, perhaps, less frequent, but two situations can be distinguished in our data. First, at least *Ceriporia tarda, Junghuhnia autumnale* and *J. fimbriatella* have continuous eastern distributions that reach Estonia, but rarely (if at all) Scandinavia. Similar species found in eastern Finland, but not in Estonia, are *Antrodia hyalina, A. tanakai,* and *Postia persicina* (Niemelä 2016). Secondly, the DNA-barcoding methods have helped us to record in Estonia *Polyporus submelanopus* and *P. ulleungus* with so-far known distributions in the Far East (Xue & Zhou 2013, Tibpromma et al. 2017). That these species have not been recorded in Europe before may reflect insufficient molecular sampling or, alternatively, natural or human-mediated long-distance dispersal. Natural cross-border immigration from Russia has been hypothesized to have caused recent population increase in Estonia in some eastern species with continuous distributions, such as *Amylocystis lapponica* (Runnel et al. 2020). In the case of Far-Eastern species there is a possibility of artificial dispersal with long-distance trade in the Soviet period of Estonia (1945–1991).

### Taxonomically unclear and exotic taxa

Taxonomically difficult situations remain common in European polypores despite much research undertaken. We documented, based on DNA (ITS) barcoding lineages, at least 20 likely undescribed species in Estonia alone. Since ITS differences can be minor among species in some genera, such as *Antrodia* and *Antrodiella* (Miettinen et al. 2012, Spirin et al. 2015), this number may increase when multiple genetic markers are used. At genus level, taxonomic revisions of *Coltricia*, *Physisporinus,* and *Sistotrema* (comprising at least 13 undescribed species in Estonia) appear as the priorities to clarify regional polypore biota. For some very rare lineages our data were too scarce to enable any ecological insight, and we encourage field work and international collaboration to add ecologically described records.

Some taxonomically resolved cases remain problematic in field sampling and for red-listing threatened species. For example, the collective taxa *Postia alni, P. caesia*, and *Skeletocutis nivea* remain in parallel use, because field identification of their cryptic constituent species is not reliable despite identification keys provided. How to apply those collective species concepts should be decided depending on questions being asked. If the goal is to record all constituent species of the collective taxa, vouchers should be regularly collected for laboratory assessment; e.g., sampling specimens from different substrates (Runnel et al. 2014).

Another uncertain part of the biota comprises exotic species. There is a considerable literature on the spread of wood-inhabiting fungi to exotic host trees, notably in plantations and on ornamental trees. Much less is known on how exotic host trees or anthropogenic substrates have changed the abundance or distribution patterns of the fungi (Burgess et al. 2016). In Estonia, parks, cemeteries, and gardens constitute poorly sampled habitats, and there are six polypore species (3% of the species pool) confined to introduced woody species in such settings. Four species are not applicable (NA) for conservation assessment: *Phellinus tuberculosus* and *Postia balsamea* have been only recorded on fruit trees in gardens, *Ganoderma carnosum* on *Abies* sp. (an exotic tree), and *Ceriporia bresadolae* on building timber. Additionally, *Phellinus hippophaeicola* has been only found once on a *Hippophae rhamnoides* (naturalized but mostly in plantations), and *Phylloporia ribis* (a frequent species) only occasionally outside gardens. A well-supported ecological conclusion, however, is that no exotic polypore has so far attained significant functional role in Estonian natural forests.

### Checklist-based detection of changes in fungal biota

Monitoring fungal diversity remains a challenge (e.g., Halme et al. 2012) and, compared with plants or animals, fungal conservation perspectives have much poorer, often indirect, background knowledge on population dynamics. Unclear background undermines using fungi as indicators, which would be reasonable for different purposes (Lonsdale et al. 2008, Junninen & Komonen 2011, Heilmann-Clausen et al. 2015). A solution has been combining ecological studies on current fungal habitat relationships with habitat changes of the past (e.g., Kouki et al. 2001, Penttilä et al. 2006, Junninen & Komonen 2011). However, this requires key factors to be well known and includes hidden assumptions of stable regional species pools and habitat relationships in time. It cannot substitute documenting of changes in fungal biota, for which unfortunately no comprehensive and feasible survey methods exist.

Updated and critically revised regional checklists that integrate multiple data sources might thus remain crucial for monitoring full fungal diversity and for red-listing threatened species (Arnolds 2001). Yet, for credible interpretation of records, checklists must incorporate quality assessment, based on intensity and distribution of sampling effort, methodological heterogeneity, and species identification methods used. A set of critical issues assessed for our study (Table 5), implies that: (i) historical changes in the Estonian polypore biota can be summarized only by individual species (total numbers of species recorded are unreliable), (ii) at the current sampling intensity, ‘safe minimum’ temporal resolution of detecting strong trends and extirpation is ca. 30 years (see below), (iii) detectability (conspicuousness; identification; ecological impact) is a key consideration for evaluating the species’ trends.

**Table 5:** A quality assessment scheme (quality criteria) proposed for regional checklists of macrofungi, exemplified by the current study

Quality criterion	Assessment for the current checklist	Limitations derived
Completeness	< 10% unrecorded valid species (estimated from Chao index based on singleton/doubleton ratio [1]; also by analyzing species recorded in neighbouring countries)	Total no. of recorded species poorly comparable
Taxonomic stability	Ca. 5% recorded species taxonomically unresolved; up to 10% further additions as currently undescribed lineages	Previous checklists cannot be used for direct comparisons
Documentation quality of source data	All collections in public fungaria; 3% with publicly accessible DNA bar-codes (incl. vouchers of most taxa). > 95% observations geo-tagged and in public databases; however, samples from ecological studies largely identified based on observations.	All species can be re-assessed from original material, but not all individuals (especially of common taxa).
Presentation quality	References to remarkable specimens and datasets presented. Difficult specimens analyzed for phylogenetic relationships. Taxonomic and ecological data linked.	Undescribed species can be followed in the material.
Differences between subsequent checklists	Within 15 yrs., 15% increase in the no. of valid species, mostly due to adding ecological sampling designs.	Different bias in historical [2] and current data (numbers of records cannot be simply corrected for sampling intensity)
Geographic coverage	Western part of the country poorly studied using ecological sampling designs.	Frequencies underestimated: taxa with western distributions.
Ecological representativeness	Important understudied habitats: naturally disturbed areas, riverine woodlands, oak stands, and wooded grasslands with ancient trees [3–4], also gardens and buildings	Frequencies underestimated: taxa inhabiting semi-open natural or cultural landscapes.
Species detectability bias	Apparent in casual collections [5]; reduced in the main ecological sampling scheme used [6].	Difficult-to-detect species poorly represented in ecosystems with casual collection data only.
e-DNA data	Not included. Extensive sequencing of soil fungi and some studies of wood samples have not revealed new species, but would probably reveal wider ecological niches of many taxa [3, 7].	Frequencies and ecological niches underestimated, specifically in mycorrhizal species.

References: [1] Chao 1987; [2] Parmasto 2004; [3] Runnel & Lõhmus 2017, [4] Lõhmus et al. 2018b, [5] Lõhmus 2009; [6] Lõhmus et al. 2018a; [7] Ovaskainen et al. 2013

Case studies illustrate these points. Regarding point (ii), a few iconic species can be perhaps monitored even at < 10 year resolution in Estonia (Runnel et al. 2020). More typically, however, a viable population of *Trametes suaveolens* (last seen in 1984 in the country) was discovered in much-visited Tallinn city in 2018; it would have been premature to consider the species Regionally Extinct (Runnel et al. 2018). Other long record gaps of rare, but apparently viable, populations include *Hapalopilus aurantiacus* and *H. ochraceolateritius* (1962–2006) and *Dichomitus squalens* (1980–2004). Highlighting point (iii), casual collection probability has varied by two orders of magnitude among Estonian polypore species, being smallest in species that produce poorly identifiable annual basidiomes (Lõhmus 2009). Such species are most likely to be missed in the country, especially if naturally rare, recently described, and inhabiting ecosystems not yet targeted by efficient ecological sampling schemes (see Lõhmus et al. 2018a). We can list around a dozen likely additions based on the well-studied Finnish biota (Niemelä 2016), e.g., *Anomoporia kamtchatica*, *Antrodia infirma* and *A. mappa*.

Considering temporal changes in the numbers of records by species (Table 2) against the study limitations (Table 5) reveals two broad patterns of change in the Estonian polypore diversity during the last 100 years. First, there is no evidence of changed total numbers of species, but apparent in the species pool is ca. 3–5% turnover (i.e., up to 10 losses and a comparable number of gains). The *losses* comprise six species officially listed as Regionally Extinct (see above) and probably a few others not encountered for decades (*Aurantiporus priscus* – since 1980, *Xanthoporus syringae* – since 1998) or unknown to have formed actual population in Estonia (*Ganoderma carnosum* – a record in 1975, *Antrodiella parasitica* – in 1995, *Perenniporia tenuis* – in 2004). All extirpated species were very rare by the twentieth century. Most reliable *gains* are among well-established conspicuous species with habitats or locations frequently visited. Such recent novelties include at least three southern species (*Coltricia cinnamomea* and *Inonotus ulmicola* first discovered in 2002, and *Trametopsis cervina* – 2015), three species with eastern distributions (*Ceriporia tarda* – 2004, *Pycnoporellus alboluteus* and *Junghuhnia autumnale* – 2010) and *Postia auricoma* (2013). Less conspicuous newcomer candidates include *Skeletocutis jelicii* (4 locations since 2015), *Hyphodontia latitans* (a single record in 1992, then 11 records since 2012) and *Gelatoporia subvermispora* (a single record in 1991, then 12 records since 2006). *Trametes gibbosa* (a southern species) may also have recently formed a true population (three locations since 2005) after a single, possibly occasional record in 1954.

Secondly, while species turnover refers to expansions and contractions of biogeographic ranges (perhaps related to climate change; cf. Musters & van Bodegom 2018), other strong trends of extant Estonian polypores suggest ecological mechanisms. Thus, no clear declines are apparent in species inhabiting common deciduous trees, including no support to Parmasto’s (2004) notes of decline in *Pycnoporus cinnabarinus* and *Trichaptum biforme*. There are some obvious increases instead, such as possibly climate-supported trends in southern species *Hyphodontia flavipora* (see also Heilmann-Clausen & Boddy 2008), *H. radula* and *Dichomitus campestris* – all formerly been considered very rare (Parmasto 2004). Increases in record numbers of less conspicuous species in similar habitats (e.g., *Antrodiella romellii* and *Ceriporia reticulata*) are rather caused by better sampling. Assuming that increased records of most inconspicuous annual polypores on strongly decayed wood follow survey effort as well, the 1–2 similar species with reductions in records may indicate actual population declines – *Porpomyces mucidus* and, perhaps, *Anomoporia bombycina*.

In conifer-inhabiting polypores, three ecological tendencies can be distinguished. Some management-sensitive species that inhabit fallen *Picea abies* trunks have increased, probably due to efforts to protect old forests. The case of *Amylocystis lapponica* is well documented (Runnel et al. 2020); other rare species with similar record patterns are *Antrodia piceata* and *Antrodiella citrinella*; and among more frequent species – *Fomitopsis rosea*, *Junghuhnia collabens,* and *Postia undosa.* Contrasting patterns, probably revealing population declines, are apparent in *Onnia leporina*, *Climacocystis borealis* and *Skeletocutis stellae*. Our data also support the decline of *Gloeophyllum abietinum* already noted by Parmasto (2004). We hypothesize that these species may be suffering from reduction of certain wood qualities, perhaps slowly grown trees (note that *O. leporina* and *C. borealis* often inhabit *Picea abies* snags). Finally, we notice increases in two formerly uncommon *Pinus*-inhabiting species that are now widespread in various forests, including extensive drained forests on former wooded mires – *Junghuhnia luteoalba* and *Skeletocutis papyracea*.

### Broad-scale ecological patterns

Ecological case studies have been crucial for quantifying local variation in populations and assemblages, e.g. revealing their impoverishment by intensive forest management and the loss of natural forest (e.g., Penttilä et al. 2006, Junninen & Komonen 2011; for Estonia, see Lõhmus 2011, Runnel & Lõhmus 2017). Our review places those findings in the context of species pools, showing eventual extirpation of some species, but also some partial recoveries in protected forests and parallel, possibly climate-driven, shifts in distribution ranges (see above). Simultaneously, the taxonomic revisions clarify confusing reports of some putative old-forest indicator species inhabiting wider forest environments in Estonia. We now know that these represent distinct taxa (such as *Antrodia cretacea* instead of *A. crassa*, and *Postia romellii* instead of *P. sericeomollis*; cf. Runnel et al. 2014 and Runnel & Lõhmus 2017), multiple species/lineages (such as among *“Hapalopilus salmonicolor”*, *Sidera vulgaris* coll., and *Physisporinus vitreus* coll.) or misidentification (*Postia lateritia*). Based on our review, ca. half of the species listed 20 years ago as old-forest (‘hemerophobic’) polypores in Estonia (Trass et al., 1999) should probably be replaced or removed from that list to keep its focus.

Our analyses of species pools indicated that, under natural conditions, polypore assemblages would mostly vary along soil conditions and dominant forest trees. This parallels with findings on soil fungi (Tedersoo et al. 2020) and implies that forestry practices that change those factors, such as draining and artificial regeneration, are likely to be highly influential to all fungi. Distinct polypore biota on calcareous soils (alvar forests) was not known before; this finding is significant because alvar forests have been heavily degraded due to historical logging and agricultural use, and they regenerate slowly after being disturbed (Laasimer 1965). Even protected alvar forests have sometimes been mismanaged by removing dead wood, which is also essential for rare bryophytes (Meier & Paal 2009).

A pattern that soil fertility can create more assemblage variation than soil moisture is not directly applicable because our analysis separated their indirect effects via tree species composition. Both effects together explain why our ordination result (Fig. 2) resembles a Cajanderian organization of forest types solely based on soil characteristics (Lõhmus 1984). At a closer look, the pattern that polypore assemblages in drained peatland forests are more similar to meso-eutrophic forests on mineral soils than to other peatland forests has not been supported for several other organism groups – draining instead appears to produce novel assemblages (Remm et al. 2013). We also acknowledge that our approach to tree species effects was simplified (three categories analysed), and future studies should better address tree-species mixtures that are typical of hemiboreal forests (see also Tedersoo et al. 2016).

The importance of soil conditions highlights a necessity to better survey soil-inhabiting polypores. Our basidiome-based datasets suggested their higher diversity in poorer site conditions that might indicate stricter resource limitation and ecological advantages for mycorrhizal life-style in poor ecosystems. In general, however, polypores are rare and unlikely at key functional positions in mycorrizal assemblages in Estonia (e.g., Tedersoo et al. 2006, 2020; Bahram et al. 2011); a possible exception is *Coltricia perennis* – a dominant colonizer of early-successional *Pinus* sites (Visser 1995, Kwaśna et al. 2019). Summarizing the work done on DNA-based soil sampling could also improve our understanding of the ecology and conservation status of several species.

Regarding substrates, we found that the species having parasitic or brown-rot decay life strategies tend to be restricted to fewer host-tree species. This is probably linked with trade-offs of these life strategies, of which better understood are the highly demanding growth conditions inside live trees that require specific stress-tolerant traits in parasites (Schwarze et al. 2013). Brown-rot fungi may have distinct physiological limitations, indicated also by their typical disability to degrade pure cellulose (Nilsson & Ginns 1979) or possibly lower wood pH optima (Highley 1976). However, these differences are in need of revision since the dichotomy of white- and brown-rot fungi has been challenged based on genetic data (Riley et al. 2014). Physiological limitations set by wood chemistry and structure and tree defence mechanisms probably explain also our finding that phylogenetically closer tree species tend to host more similar pools of polypore species. Some ecological confounding effects are possible (i.e., related tree species may also grow in similar sites) but not very likely, given our result of the similarity of polypore assemblages in the hydrologically contrasting dry boreal and bog sites (both dominated by *Pinus sylvestris*).

Comparison of species pools on different woody substrates reveals an unexpected issue with natural stand regeneration – a sustainability indicator in forestry (Forest Europe, 2015). In the Estonian clear-cutting based forestry, natural regeneration on fertile sites mostly comprises *Betula*, *Alnus*, and *Populus* species, which cluster together by polypore assemblages (Fig. 5). Planting *Picea abies* may diversify this situation if the stands are allowed to develop into mixed stands with coarse woody debris present (Lõhmus 2011), while the third cluster of broad-leaved trees would still be absent. Given also that *Picea abies* hosts the most diverse polypore assemblages overall (Table 4), of which large part inhabits old stands (Runnel & Lõhmus 2017), there is clear conservation motivation to use silvicultural alternatives that better account for substrate diversity (see also Lõhmus et al. 2018b). We also noticed that *Fraxinus* and *Ulmus*, both currently suffering from dieback due to introduced pathogens in Europe (Brasier 1991, Pautasso et al. 2013), have only moderately distinct assemblages when the remaining native tree diversity is present (Fig. 5). Thus, these specific dieback episodes are not likely to have strong negative impact on polypore biota in Estonia.

### A perspective

Our broad question was whether, in the case of fungi, critically appraised checklists might provide standard input to global biodiversity indicators, and whether polypores could constitute a fungal group to be included. Looking at the insights obtained in Estonia, we consider this a promising direction, which depends on standardizing checklist quality, attaining a representative sample of checklists from different parts of the world, and including ecological data. Among potential values of such a scheme would be inclusion of many rare species and utilizing historical information. The possibility for a retrospective might even be a criterion for including fungal groups (e.g., epiphytic lichens; Ellis et al. 2011). However, it is unlikely that current monitoring and retrospectives can use similar methods, which again points at checklists as a common platform. We thus encourage new regional syntheses on polypores and other long-studied fungal groups.

## CONCLUSION

Our review demonstrates how integrating multiple data sources and their taxonomic and ecological appraisal can provide new perspectives on fungal species pools, rare and undescribed species, and their long-term dynamics. The test case, the Estonian polypore biota, comprises over 260 species, of which 221 are verified extant species, and the remaining are molecularly documented but yet-undescribed lineages or species probably present but remaining to be found. During the last 100 years, the biota experienced ca. 3–5% species turnover, including directional changes but no obvious trend in diversity. Attaining a representative sample of high-quality checklists for flagship fungal groups from could be an approach to elaborating global indicators of fungal diversity.

## Supplementary Information

**Additional file 1.** Estonian forest types, their main characteristics, and treatment in the polypore habitat analyses.

**Additional file 2.** The 2-ha plots of systematic polypore surveys in Estonia, their woodland type classifications and references to publications that used the survey results.

**Additional file 3.** Collection details, UNITE or GenBank accession numbers for ITS and LSU sequences of Estonian specimens analyzed in this study.

**Additional file 4.** Specimen vouchers, geographic location, and UNITE or GenBank accession numbers for public reference sequences (ITS and LSU) used in phylogenetic trees.

**Additional file 5.** Taxonomic notes and phylogenetic trees of difficult species.

**Additional file 6. **Non-metric multidimentional scaling (NMDS) ordination diagrams of polypore assemblages: (A) in forests on fertile and poor (excl. calcareous) soils and thin calcareous soils; (B) in woodlands with *Picea* and *Pinus* (including their mixedwood) or dominated by deciduous trees.

**Additional file 7.** Numbers of common and unique species for habitat combinations not shown on Euler diagrams (Fig. 3).

**Additional file 8.** Nutritional-mode categorization of wood-inhabing polypore species with > 10 records in Estonia (input data for Fig. 4).

## Data Availability

The datasets generated and/or analysed during the current study are available in the Plutof repository: https://plutof.ut.ee/#/doi/10.15156/BIO/786358https://plutof.ut.ee/#/doi/10.15156/BIO/786363 https://plutof.ut.ee/#/doi/10.15156/BIO/786357

## Abbreviations

DNA: Deoxyribonucleic acid
rDNA: Ribosomal ribonucleic acid
ITS: Internal transcribed spacer
LSU: Large subunit
MRPP: Multi-response permutation procedures
NMDS: Non-metric multidimensional scaling
