# Supplementary material for: Polypore fungi as a flagship group to indicate changes in biodiversity – a test case from Estonia
Source: IMA Fungus. 2021 Jan 18;12:2. doi: 10.1186/s43008-020-00050-y (PMC7812660; doi:10.1186/s43008-020-00050-y)
Supplement: Supplementary file 5 — Additional file 5. Taxonomic notes and phylogenetic trees of difficult species. [file 43008_2020_50_MOESM5_ESM.docx]

# ***Additional file 5. Taxonomic notes and phylogenetic trees of difficult species***

Polypore fungi as a flagship group to indicate changes in biodiversity – a test case from Estonia. Runnel K, Miettinen O, Lõhmus A. Corresponding author: Kadri Runnel, Tartu University, kadri.runnel@ut.ee

***Antrodiella faginea****: Antrodiella* species are often difficult to separate morphologically because of their small basidiomes and uniform microscopy (Miettinen et al. 2006); they also appear genetically close (interspecific variation in the ITS regions only 1–2 base pairs or 0.2%) (Miettinen et al. 2012). Based on the ITS regions, the Estonian specimens macro- and micromorphologically identified as *A. faginea* represent two lineages, which both differ from the sequence from the type (AF126884, Fig. A5-1, Table 3) and appear closely related to the North-American *A. semisupina*. The Estonian lineages inhabit dead wood of several deciduous tree species in various managed forests, but both are infrequent (four and seven molecularly confirmed specimens, respectively). Because of a lack of apparent conservation concern (wide habitat niche; few records perhaps due to low detectability – see Lõhmus 2009), the lineages can be considered a collective species in biodiversity assessments.


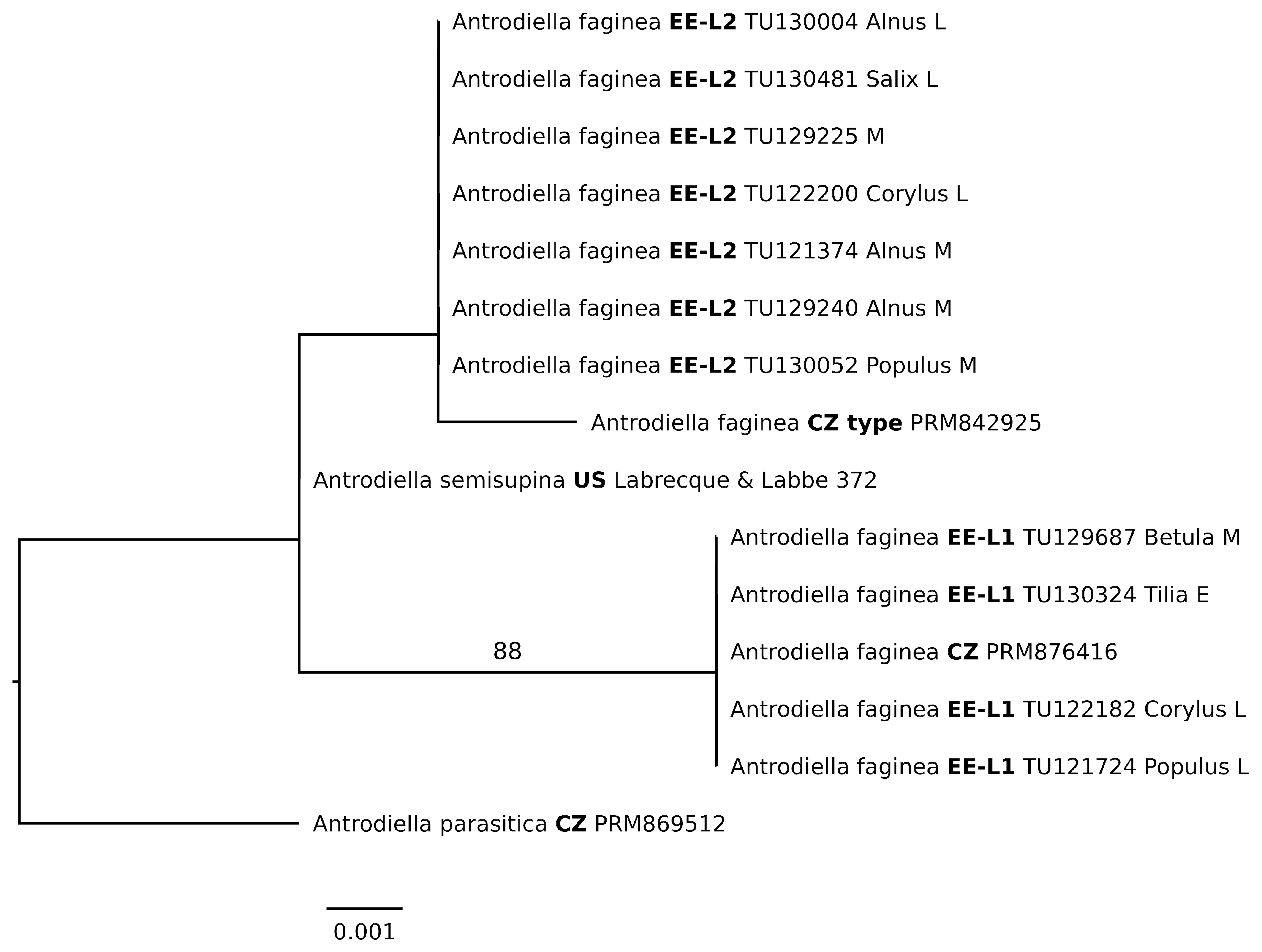


**Fig. A5-1.** ML phylogeny (ITS sequences) of Estonian samples of *Antrodiella faginea* *sensu* *lato* from different successional stages (E, early successional; M, mature stands; O, old growth) and host tree species; and their closest references from public databases. Based on Johannesson et al. (2000), the tree was rooted to *Antrodiella parasitica*. Numbers on nodes represent ML bootstrap values >70%, the scale bar indicates the number of expected substitutions per site. The analysis was performed, using K2P substitution model.

***Byssoporia terrestris***: A rare species in Estonia and in Northern Europe (Josefsson & Spirin 2010), which lacks DNA-based taxonomic and ecological assessment. The three sequenced specimens from Estonia, macro- and micromorphologically identified as *B. terrestris*, represent two distinct lineages with highly variable ITS regions (Table 3). The ITS sequence for the Estonian lineage 1 is almost identical to *B. terrestris* sequences from Sweden and Finland; whereas lineage 2 lacks close matches in the reference databases, but clusters with a *B. terrestris* sequence from Sweden (Fig. A5-2).


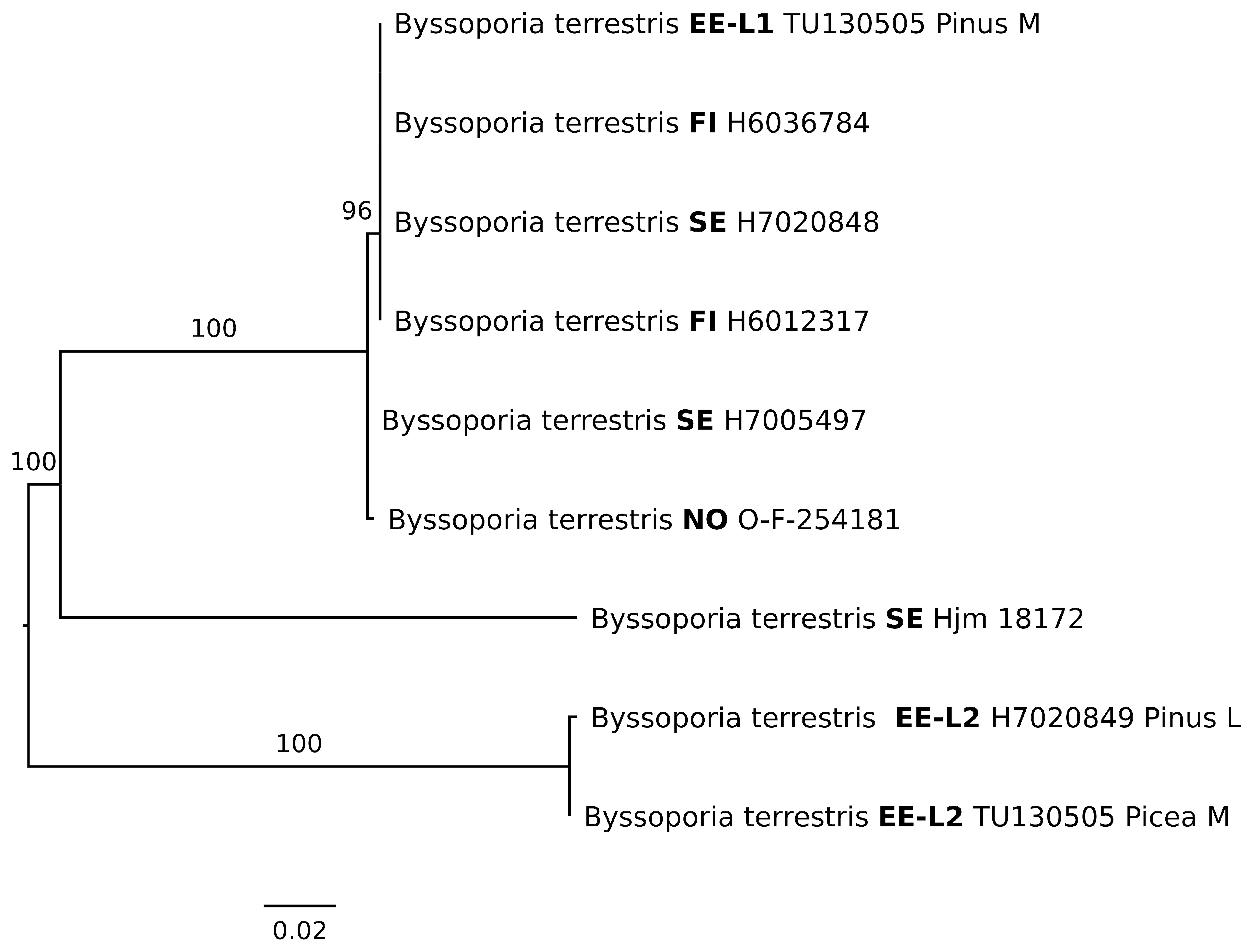


**Fig. A5-2**. ML phylogeny (ITS sequences) of Estonian samples of *Byssoporia terrestris* *sensu lato*; and their closest references from public databases. The tree was centrally rooted and the analysis based on K2P+I substitution model. See Fig. A5-1 for the symbols and abbreviations.

***Ceriporia* spp.**: The species in *Ceriporia* (except the well-established and distinct *C. reticulata*) pose both taxonomic and conservation questions. The Estonian data reveal six such *Ceriporia* species, divided between two taxonomic groups. Since both groups comprise rare species that are difficult to recognize in the field, we recommend collecting vouchers of all yellow, purple, and red specimens in the field. The *C. purpurea* group comprises *C. purpurea* and two kin species: the recently described *C. torpida* and re-introduced name *C. bresadolae* (Spirin et al. 2016). This group is of conservation interest: all the species appear extremely rare and are ecologically poorly known.

The *C. viridans* group poses both conservation and taxonomic challenges: it comprises, according to our molecular data, *C. viridans* *s. str.* (common: >50 records), *C. aurantiocarnescens* (rare: 5 records), *C. excelsa* (common: >50 records), and two unnamed lineages. The ITS-LSU sequence of one unnamed lineage clusters with *C. viridans s. str.* in ML analyses (*C. viridans* L2 and L1, respectively in Fig. A5-3), but lacks close matches in reference databases. This lineage is repeatedly found on various tree species and forest successional stages in Estonia (Table 3) and can be considered a collective species with *C. viridans s. str.* in biodiversity assessments. The other undescribed lineage clusters with *C. excelsa* sequences from Estonia and Norway and is almost identical to a GenBank reference of a North-American *Ceriporia* sp. It is represented by a sole Estonian collection (TU124431) in a mixed boreo-nemoral old-growth forest (a well sampled habitat); this is therefore most probably a rare taxon of conservation concern.


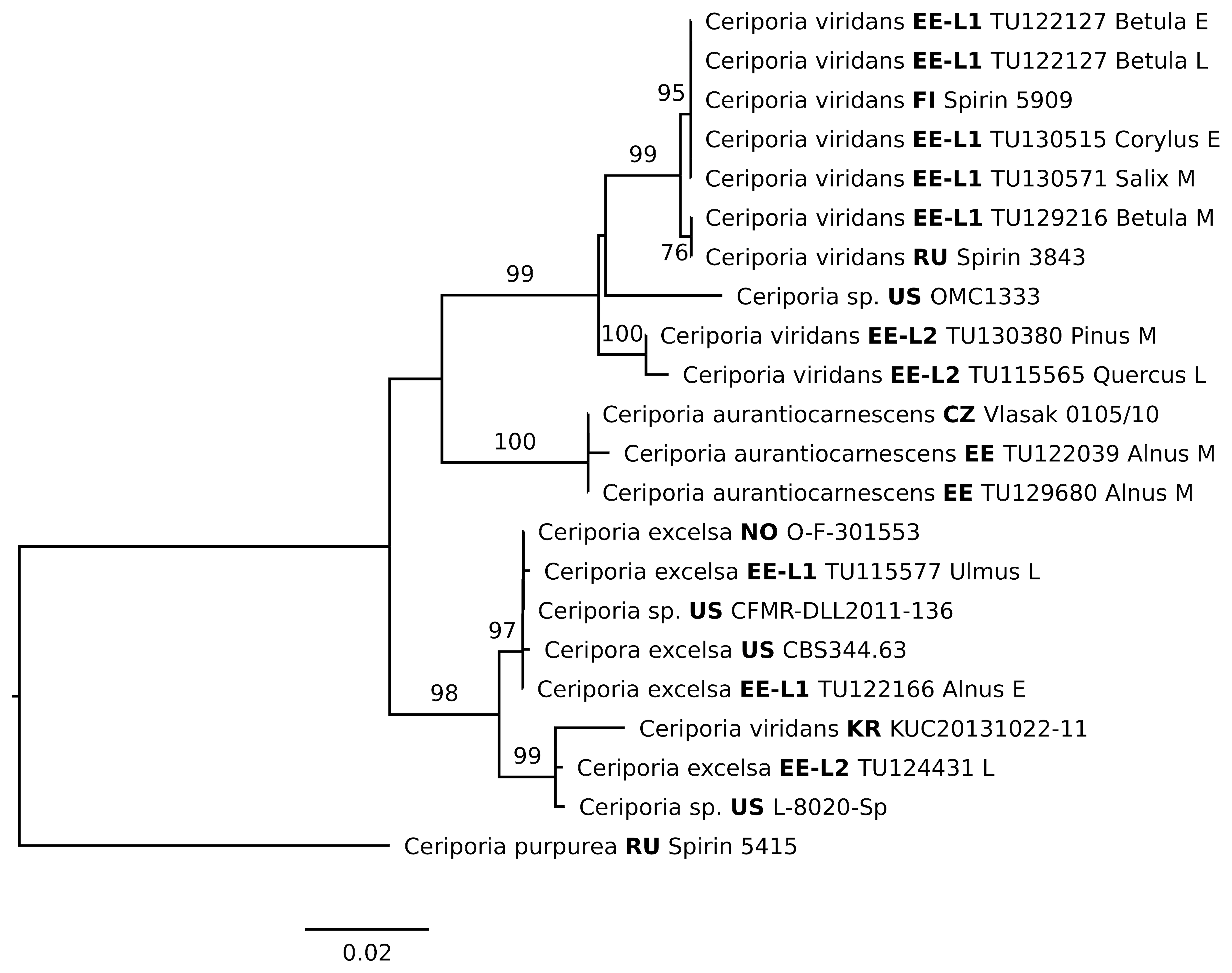


**Fig. A5-3**. ML phylogeny (ITS+LSU sequences) of Estonian samples of *Ceriporia viridans* group from different habitats and host tree species; and their closest references from public databases. Based on Spirin et al. (2016), the tree was rooted to *Ceriporia purpurea*. The analysis was performed using TPM2u+F+R2 substitution model. See Fig. A5-1 for the symbols and abbreviations.

***Ceriporiopsis pseudogilvescens* and *C. resinascens*:** ITS sequences from Estonia support the distinction of the closely related *Ceriporiopsis pseudogilvescens* and *C. resinascens* (12 and 5 sequenced collections, respectively) (see also Kinnunen & Niemelä 2005, Tomšovský et al. 2010). Additionally, a third Estonian lineage with small, but consistent, differences in ITS can be distinguished (2 sequenced collections) (Table 3) and at least five sequenced specimens have ITS copies from several above-mentioned lineages (sequence data in Additional file 3). The main morphological characteristics (colour, pore, and spore size) represent a continuum and cannot be used to reliably distinguish the lineages in this complex; there are also no clear habitat differences.

***Coltricia cinnamomea* and *C. perennis*:** The Estonian specimens macro- and micromorphologically identified as *Coltricia cinnamomea* and *C. perennis* have highly variable ITS regions: each represents at least three lineages (Figs A5-4and A5-5). *C. perennis* appears paraphyletic as one lineage (TU106860) clusters together with the closely related *C. confluens* (found only twice in Estonia) (Fig. A5-5). Among *C. cinnamomea*, two lineages cluster together with *C. cinnamomea* from China, whereas one clusters together with *C. subperennis* known from China (Fig. A5-4). Both collective species are of conservation interest: *C. cinnamomea* as a rare old-forest species (four of the six records from coastal old-growth stands in Estonia) with the extinction risk of individual lineages apparently even higher than current Endangered status. *C. perennis* has been nationally assessed as of Least Concern (also elsewhere in Europe), but some of its lineages may be threatened due to rarity or specific habitat requirements.


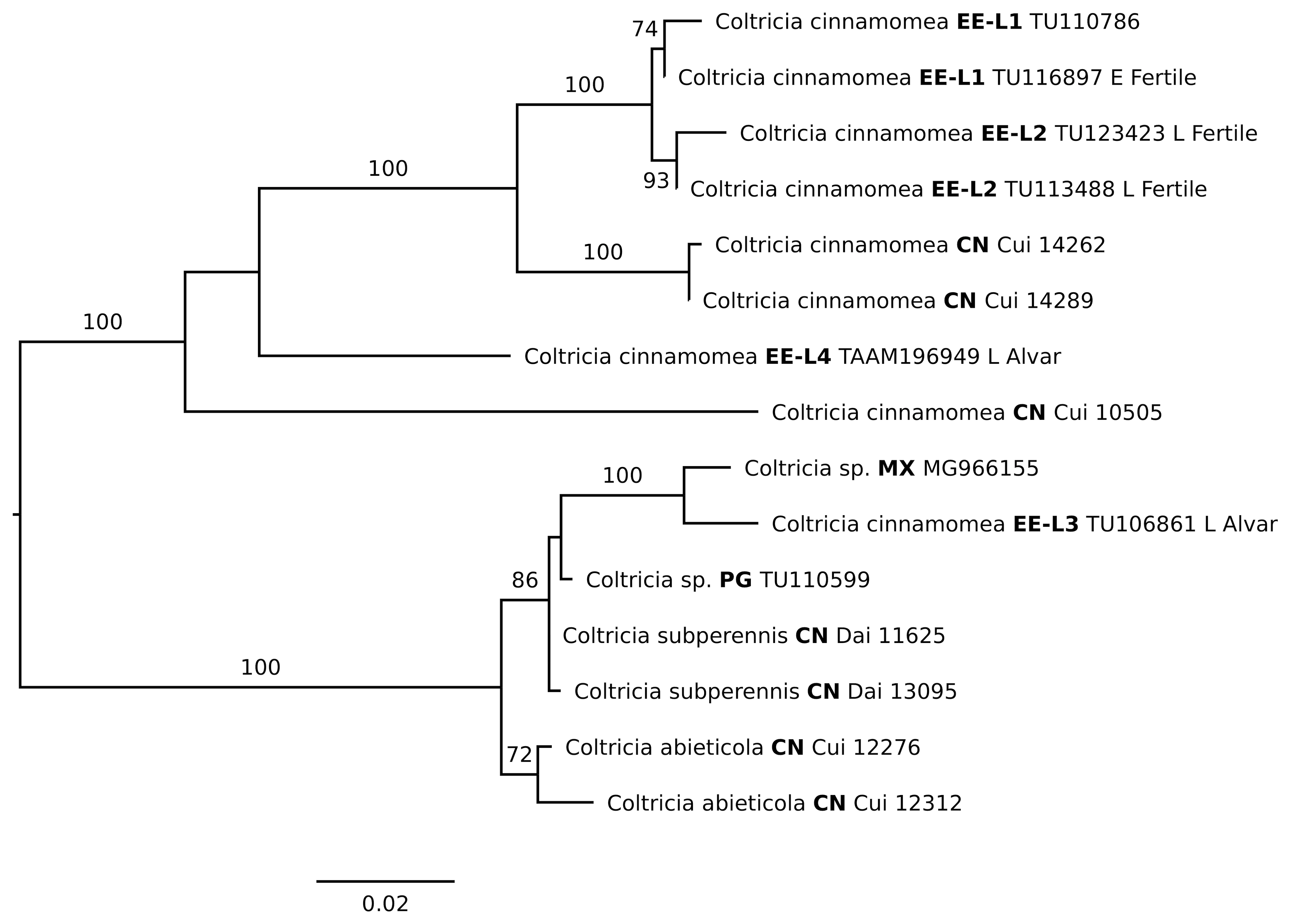


**Fig. A5-4.** ML phylogeny (ITS sequences) of Estonian samples of *Coltricia cinnamomea* from different sites (alvar, poor- and fertile-soil) and forest successional stages (E, early successional; M, mature stands; O, old growth); and their closest references from public databases. The tree was centrally rooted and the analysis performed using HKY+F+G4 substitution model.


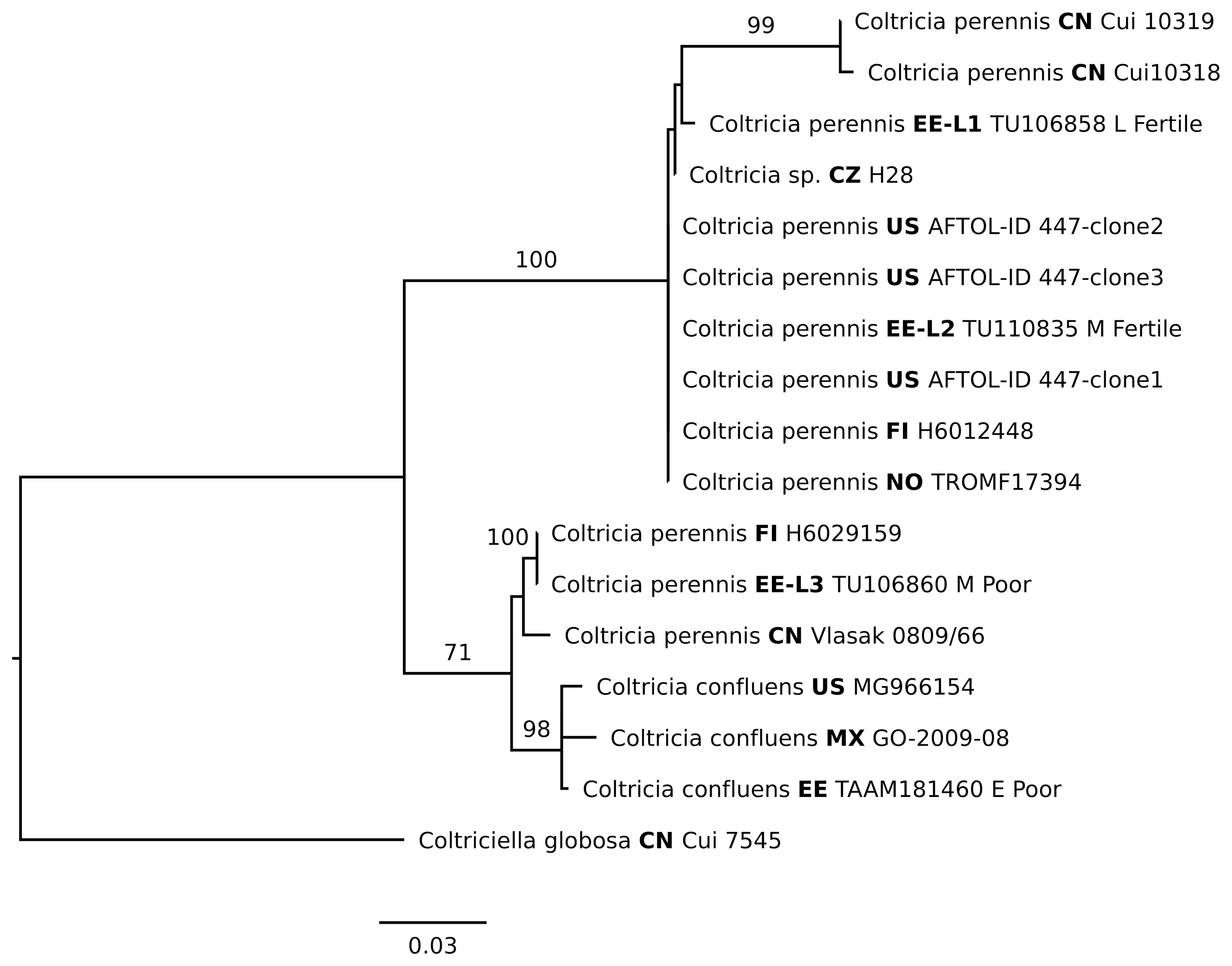


**Fig. A5-5.** ML phylogeny (ITS sequences) of Estonian samples of *Coltricia perennis* from different habitats and host tree species; and their closest references from public databases. Based on Bian et al. (2016), the tree was rooted to *Coltriciella globosa*. The analysis was performed using HKY+F substitution model. See Fig. A5-4 for the symbols and abbreviations.

***Physisporinus* spp.:** Phylogenetic relationships between morphologically similar resupinate species of *Physisporinus* remain unclear. The Estonian ITS-sequence data show three currently accepted species in this group: *P. sanguinolentus, P. vitreus,* and *Rigidoporus undatus* (syn. *Physisporinus undatus*)*,* all requiring further work. *P. vitreus* is used as an indicator species of nationally valuable forest habitats (Riigi Teataja 2017), but appears unsuitable for such use due to unresolved taxonomy and unclear ecological requirements.

Both *P. sanguinolentus* and *P. vitreus* are collective species. In *P. sanguinolentus*, the common lineage 1 (>100 records) agrees with the rather uniform concept of this species in Europe. A distinct lineage 2 (Table 3) has been collected only once and has no close references from elsewhere in Europe; it appears closely related to *P.* *furcatus* (syn. *Rigidoporus*) samples from China, but differs still by 14 base pairs from the sequence of the type collection of *P. furcatus* from the Russian Far-East (TAA-15097, Fig. A5-6). The Estonian lineages of *P. vitreus* *s. lat.* have distinct ecologies and abundances. Lineages 1–3 (Fig. A5-6, Table 3) appear all closely related to *P. tibeticus* from China and are common (>50 confirmed records) in a specific habitat: waterlogged spots, such as under fallen deciduous trunks and branches on mud. A distinct lineage 4 (Fig. A5-6, Table 3) is rare in Estonia (four confirmed records), and also for example the Czech Republic, but is common in Finland (Otto Miettinen, unpubl.); it may represent a northerly restricted species. Lineage 4 is found in diverse forests on both coniferous and deciduous hosts.

The records are insufficient for assessing population status of *P. undatus* in Estonia.


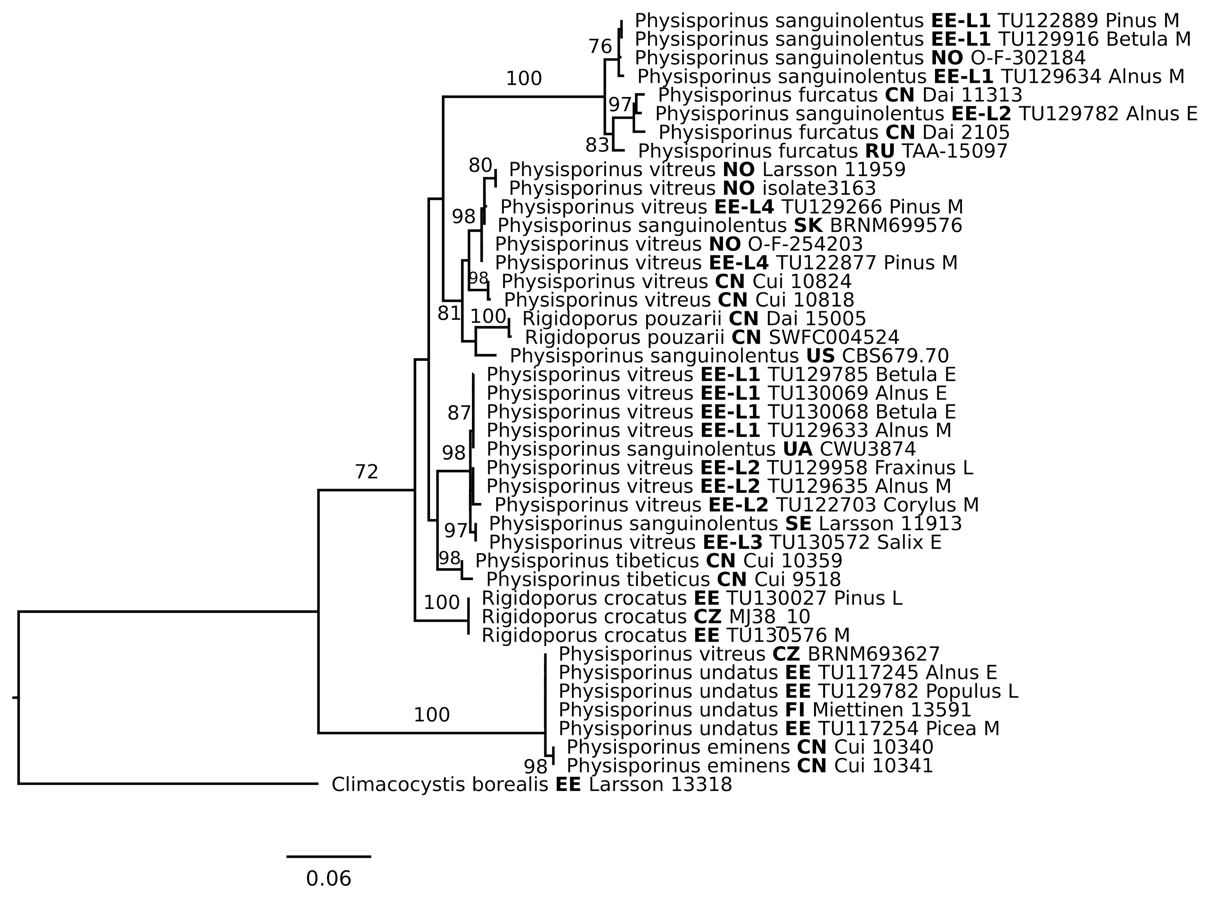


**Fig. A5-6.** ML phylogeny (ITS sequences) of the Estonian samples of *Physisporinus* spp. from different habitats and host tree species; and their closest references from public databases. Based on Justo et al. (2017), the tree was rooted to *Climacocystis borealis*. The analysis was performed using HKY+F+G4 substitution model. See Fig. A5-1 for the symbols and abbreviations.

**Black-stiped *Polyporus* spp.:** Four black-stiped *Polyporus* species have been recognized in hemiboreal Europe: *P. badius, P. leptocephalus, P. melanopus,* and *P. tubaeformis* (e.g., Niemelä 2016). The sequenced material from Estonia reveals two more species recently described from Asia and not previously reported in Europe: *P. submelanopus* and *P. ulleungus* (both recorded once; reference data in Additional file 3)*. P. submelanopus* was found on an unknown substrate in a wooded meadow on Hiiumaa Island, and *P. ulleungus* on a fallen *Betula* trunk in an 80 year-old boreo-nemoral eutrophic forest in inland Estonia. Also, *P. melanopus* and *P. tubaeformis* appear as rare, but these species may be under-recorded as their ecology is poorly known. The label data on historical fungarium specimens does not reveal clear habitat patterns and can include within-group misidentifications. Despite abundant substrate (dead wood of common deciduous trees), only *P. leptocephalus* has been found during extensive systematic surveys in forests (dataset II in Table 1). *P. melanopus* and *P. tubaeformis* have been recorded in various forests and parks, with no clear geographical pattern in Estonia.

***Postia leucomallella*:** *P. leucomallella* represents two cryptic species, *P. calvenda* and *P. rufescens ad int.* (Niemelä 2016), which are both present among Estonian collections as confirmed by both morphological and molecular data (reference data in Additional file 3). The collective species has been considered well identifiable previously, and most Estonian data are thus field observations that cannot be tracked back to one of the constituent species. *P. rufescens* appears to be common and comprises *ca*. 90% of checked collections in Estonia. The data are insufficient for ecological description, but no old-forest association is evident in either species.

***Postia caesia* group:** Recently, Miettinen et al. (2018) revised the taxonomy of the *Postia caesia* group and divided the two species earlier known as *P. alni* and *P. caesia* into seven species in Northern Europe (*P. alni*, *P. caesia*, *P. caesiosimulans, P. cyanescens,* *P. populi*, *P. subcaesia,* and *P. simulans*). Since the host tree species was previously considered (also by Parmasto 2004) rather reliable for the field separation of *P. alni s. lat.* (on deciduous trees) and *P. caesia s. lat.* (on conifers), the Estonian data cannot be readily re-assessed for the newly separated species. Our molecular data of the few collected and sequenced specimens revealed the presence of *P. alni*, *P. caesia*, *P. cyanescens,* and *P. simulans* in Estonia (reference data in Additional file 3). The data reveal that, in Estonia, *P. alni* produces basidomes also on *Populus tremula,* and *P. cyanescens* also on *Pinus sylvestris* (a rare host; cf. Miettinen et al. 2018).

**Poroid *Sidera* spp.:** This recently described genus (Miettinen & Larsson 2011) comprises two dimitic polypores: the perennial *Sidera lenis* and the annual *S. vulgaris* (possibly a collective species). Niemelä (2016) unofficially introduced another perennial species designation, *Sidera mitis ad int.,* – a *Picea*-dwelling kin of *S. lenis.* The Estonian sequence data confirm at least three poroid *Sidera*-species as present in Estonia (Table 3, Fig. A5-7): two annual lineages (*S. vulgaris* coll.), and a perennial lineage, which does not match with the prevailing species concept of *S. lenis* (but rather corresponds to *S. mitis* *ad int.*). The latter inhabits *Picea*, *Pinus,* and *Alnus,* and may be a conservation dependent old-forest species (all the Estonian records with habitat data are from old growth forests). The annual lineages may have contrasting conservation status; the ITS sequences of lineage 1 (Table 3) cluster with *S. lenis* from Finland and *S. vulgaris* from New Zealand. It appears rare and can be threatened: both Estonian collections were on slowly grown *Pinus sylvestris* trunks in forested wetlands. The annual lineage 2 forms a well-supported clade and appears closely related to a *Sidera* sp. from the USA; that lineage is frequent on several host-trees and forest successional stages in Estonia (>50 confirmed records).


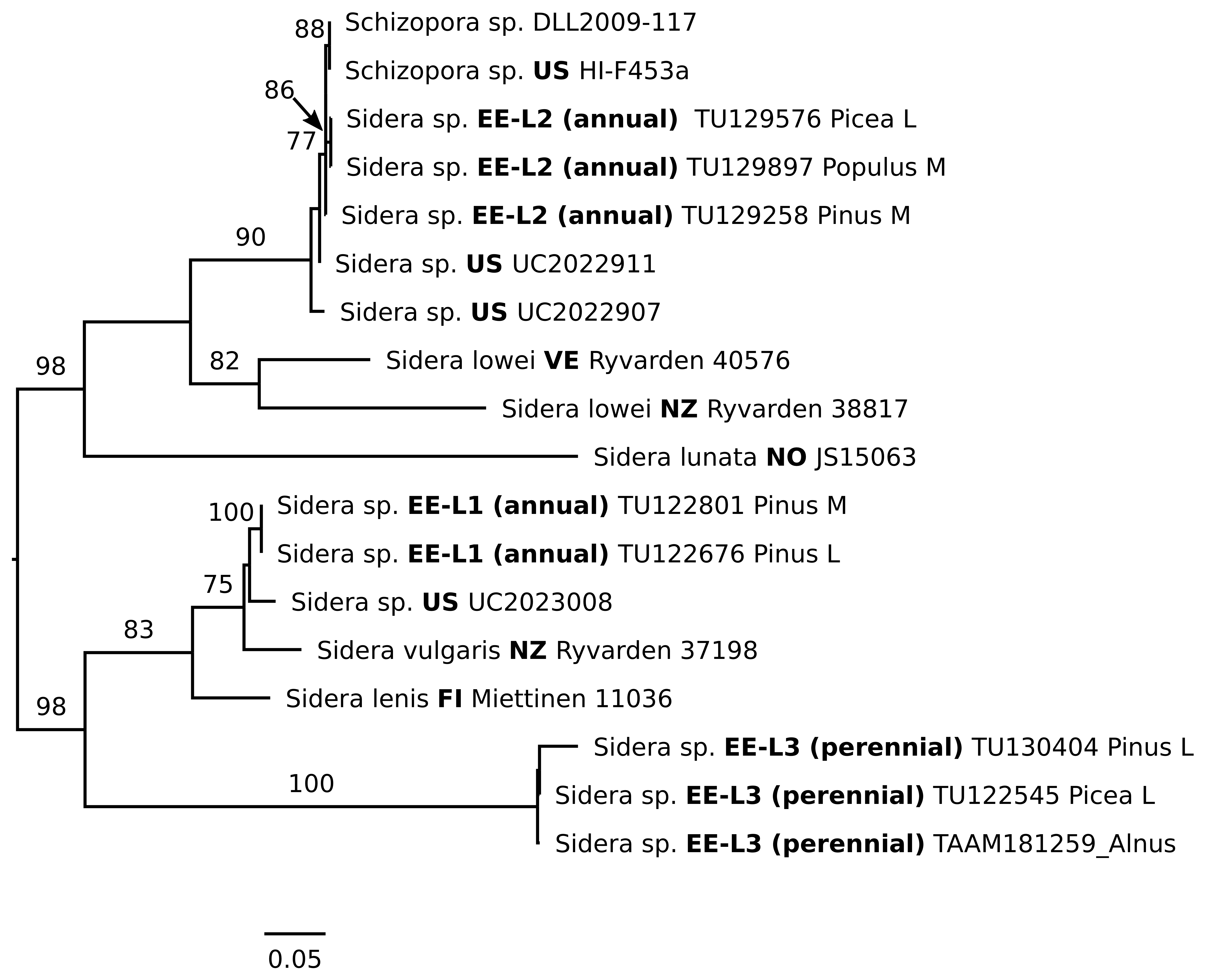


**Fig. A5-7.** ML phylogeny (ITS+LSU sequences) of Estonian lineages of poroid species in *Sidera* spp. from different habitats and host tree species; and their closest references from public databases. The tree was centrally rooted and the analysis performed using TNe+G4 substitution model. See Fig. A5-1 for the symbols and abbreviations.

**Poroid *Sistotrema* spp.:** Several studies show (Nilsson et al. 2006) or suggest (Di Marino et al. 2008, Niemelä 2016) that, among the two European poroid *Sistotrema* species, *S. muscicola* is a collective species, while its kin species *S. alboluteum* has hithertho been considered sound. Our ITS sequence data reveal that the prevailing morphology (spore size) based species concepts of both *S. muscicola* and *S. alboluteum* are non-monophyletic, the first comprising at least four and the second at least two molecularly distinct lineages (Fig. A5-8). *S. muscicola s. lat.* has been found on several tree species and substrate types in dry conifer dominated forests and is currently of no conservation concern, while *S. alboluteum* appears rarer and restricted to conifers.


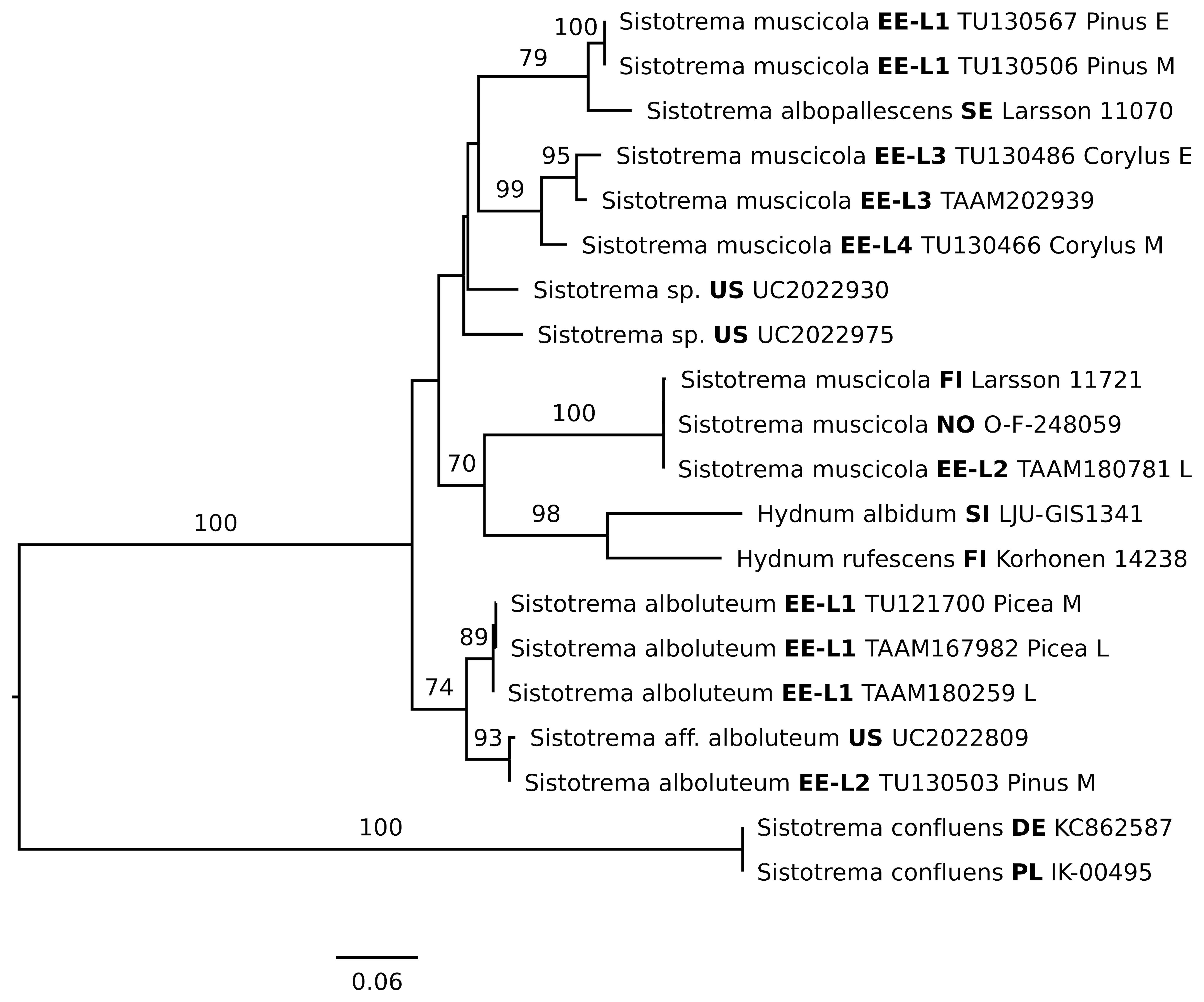


**Fig. A5-8**. ML phylogeny (ITS sequences) of Estonian lineages of poroid species in *Sistotrema* spp. from different habitats and host tree species; and their closest references from public databases. The tree was centrally rooted and the analysis performed using HKY+F+G4 substitution model. See Fig. A5-1 for the symbols and abbreviations.

***Skeletocutis kuehneri* / *S. brevispora* group:** Miettinen & Niemelä (2018) recently revised the taxonomy of resupinate polypores closely related to *Skeletocutis kuehneri* and *S. brevispora*, and described two new species in this group (*S. exilis* and *S. delicata*). The latter are both present in Estonia (Fig. A5-9), and at least *S. delicata* deserves conservation attention as a follower species of another threatened species (*Phellinus ferrugineofuscus*). Our data indicate further undescribed diversity: the ITS sequence of TU128024, from a eutrophic primeval-forest remnant, clusters with the *S. kuehneri* / *S. brevispora* group but is morphologically distinct (the colour turns brown when bruised). Estonian old-growth forests on fertile soils are well surveyed, hence TU128024 may represent a specialised and threatened but undescribed species.


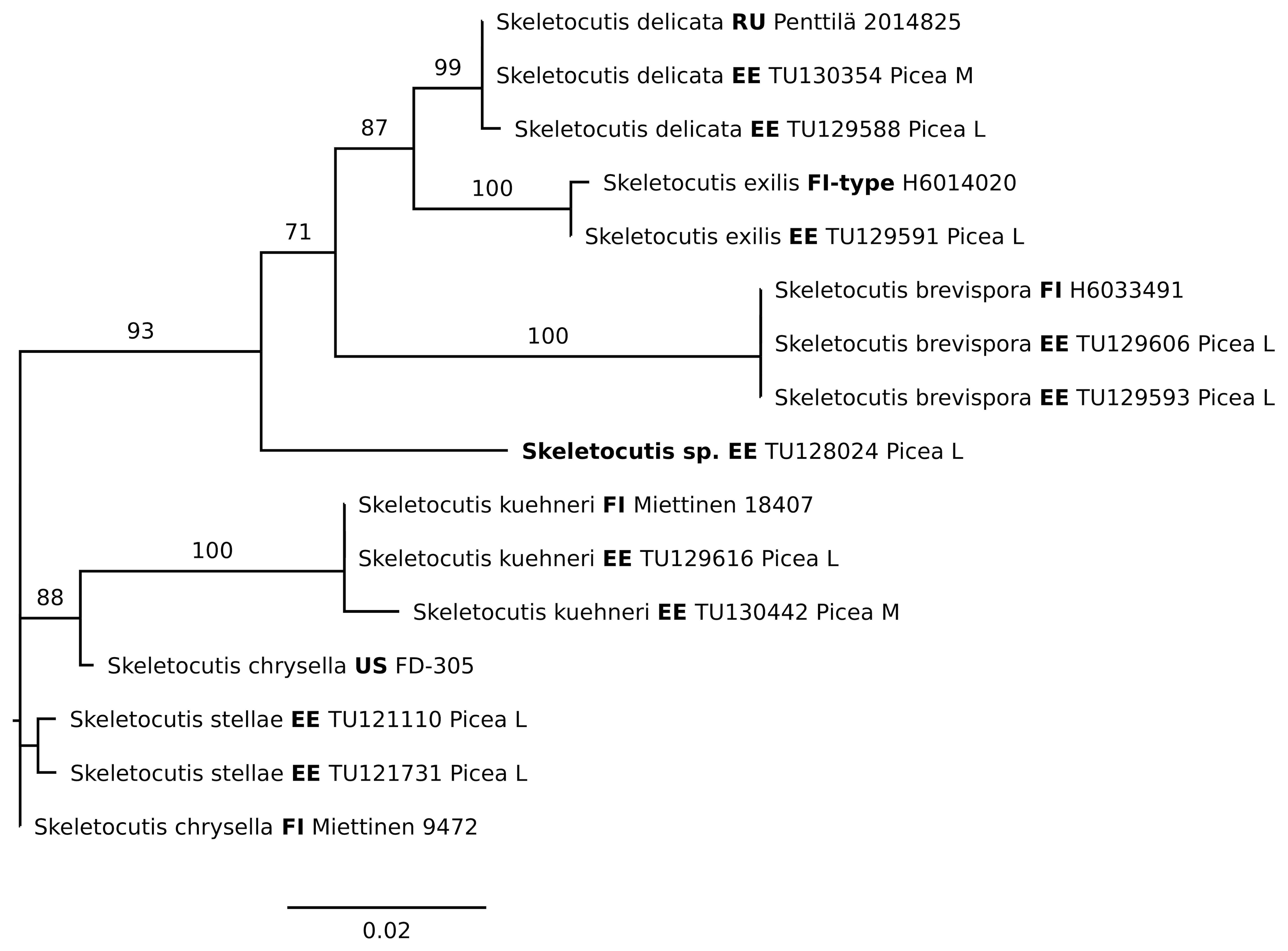


**Fig. A5-9.** ML phylogeny (ITS sequences) of Estonian lineages and species in *Skeletocutis brevispora / kuehneri* group from different habitats and host tree species; and their closest references from public databases. Based on Miettinen & Niemelä (2018), the tree was rooted to *Skeletocutis chrysella*. The analysis was performed using HKY+F+I substitution model. See Fig. A5-1 for the symbols and abbreviations.

***Skeletocutis nivea* group:** Recently, Korhonen et al. (2018) split the species inhabiting deciduous trees and earlier treated as *Skeletocutis nivea* (also in Parmasto 2004) to three species in Europe: *S. futilis*, *S. nemoralis,* and *S. semipileata*. Of these, only *S. futilis* can be separated morphologically by spore size*.* Our ITS sequences show that all these three species occur in Estonia (not visualised; reference data in Additional file 3), but the data are insufficient to assess whether any of these is threatened. Another problem is that we have one *S. nemoralis* record on *Picea abies* (misidentified by Lõhmus 2011 as *S. cummata*, earlier known as *S. ochroalba*). In this group, only *S. cummata* was so far known from *Picea* in the region (Korhonen et al. 2018). *S. cummata* is a rare and regionally threatened species (Endangered in Estonia; Vulnerable in Sweden; ArtDatabanken 2015), which is morphologically difficult to distinguish from its deciduous-tree dwelling kins and in field mostly identified based on the host species. Our observation implies that thorough morphological examination or molecular analyses are also required for *S. cummata* (it can be even more threatened than currently known).

# ***References***

ArtDatabanken (2015) The 2015 Swedish Red List. ArtDatabanken SLU, Uppsala.

Di Marino E, Scattolin L, Bodensteiner P, Agerer R (2008) *Sistotrema* is a genus with ectomycorrhizal species− confirmation of what sequence studies already suggested. Mycological Progress 7:169–176.

Bian LS, Wu F, Dai YC (2016) Two new species of Coltricia (Hymenochaetaceae, Basidiomycota) from southern China based on evidence from morphology and DNA sequence data. Mycological Progress 15:27.

Durkin L, Jansson T, Sanchez M, Khomich M, Ryberg M, Kristiansson E, Nilsson RH (2020) When mycologists describe new species, not all relevant information is provided (clearly enough). MycoKeys:72–109.

Johannesson H, Renvall P, Stenlid J (2000) Taxonomy of Antrodiella inferred from morphological and molecular data. Mycological Research 104:92–99.

Josefsson T, Spirin VA (2010) Records of rare aphyllophoroid fungi on Scots pine in northern Sweden. Karstenia 50:45–52.

Justo A, Miettinen O, Floudas D, Ortiz-Santana B, Sjökvist E, Lindner D, Nakasone K, Niemelä T, Larsson KH, Ryvarden L, Hibbett DS (2017) A revised family-level classification of the Polyporales (Basidiomycota). Fungal Biology 121:798–824.

Kinnunen J, Niemelä T (2005) North European species of *Ceriporiopsis* (*Basidiomycota*) and their Asian relatives. Karstenia 45:81–90.

Korhonen A, Seelan JSS, Miettinen O (2018) Cryptic species diversity in polypores: the *Skeletocutis* *nivea* species complex. MycoKeys 36:45–82.

Lõhmus A (2009) Factors of species-specific detectability in conservation assessments of poorly studied taxa: the case of polypore fungi. Biological Conservation 142:2792–2796.

Lõhmus A (2011) Silviculture as a disturbance regime: the effects of clear-cutting, planting and thinning on polypore communities in mixed forests. Journal of Forest Research 16:194–202.

Miettinen O, Larsson E, Sjökvist E, Larsson KH (2012) Comprehensive taxon sampling reveals unaccounted diversity and morphological plasticity in a group of dimitic polypores (*Polyporales*, *Basidiomycota*). Cladistics 28:251–270.

Miettinen O, Larsson KH (2011) *Sidera*, a new genus in *Hymenochaetales* with poroid and hydnoid species. Mycological Progress 10:131–141.

Miettinen O, Niemelä T (2018) Two new temperate polypore species of *Skeletocutis* (*Polyporales*, *Basidiomycota*). Annales Botanici Fennici 55:195–206.

Miettinen O, Niemelä T, Spirin W (2006) Northern *Antrodiella* species. Mycotaxon 96:211–239.

Miettinen O, Vlasák J, Rivoire B, Spirin V (2018) *Postia caesia* complex (*Polyporales*, *Basidiomycota*) in temperate Northern Hemisphere. Fungal Systematics and Evolution 1:101-129.

Niemelä T (2016) Suomen käävät [The polypores of Finland]. Norrlinia 31:1–430.

Nilsson RH, Larsson KH, Larsson E, Kõljalg U (2006) Fruiting body-guided molecular identification of root-tip mantle mycelia provides strong indications of ectomycorrhizal associations in two species of *Sistotrema* (*Basidiomycota*). Mycological Research 110:1426–1432.

Parmasto E (2004) Distribution maps of Estonian fungi, III. Pore fungi. Institute of Zoology and Botany of the Estonian Agricultural University, Tartu.

Riigi Teataja (2017) Vääriselupaiga klassifikaator, valiku juhend, vääriselupaiga kaitseks lepingu sõlmimine ja vääriselupaiga kasutusõiguse arvutamise täpsustatud alused. https://www.riigiteataja.ee/akt/116122010003 (accessed 31.07.2019).

Spirin V, Vlasák J, Rivoire B, Kout J, Kotiranta H, Miettinen O (2016) Studies in the *Ceriporia purpurea* group (*Polyporales*, *Basidiomycota*), with notes on *Ceriporia* species. Cryptogamie Mycologie 37:255–258.

Tomšovský M, Menkis A, Vasaitis R (2010) Phylogenetic relationships in European *Ceriporiopsis* species inferred from nuclear and mitochondrial ribosomal DNA sequences. Fungal Biology 114:350–358.
