# Supplementary material for: Polypore fungi as a flagship group to indicate changes in biodiversity – a test case from Estonia
Source: IMA Fungus. 2021 Jan 18;12:2. doi: 10.1186/s43008-020-00050-y (PMC7812660; doi:10.1186/s43008-020-00050-y)

**Additional file 6.** Non-metric multidimentional scaling (NMDS) ordination diagrams of polypore assemblages: (A) in forests on fertile and poor (excl. calcareous) soils and thin calcareous soils; (B) in woodlands with *Picea* and *Pinus* (including their mixedwood) or dominated by deciduous trees.

Polypore fungi as a flagship group to indicate changes in biodiversity – a test case from Estonia.

Runnel K, Miettinen O, Lõhmus A. Corresponding author: Kadri Runnel, Tartu University, kadri.runnel@ut.ee


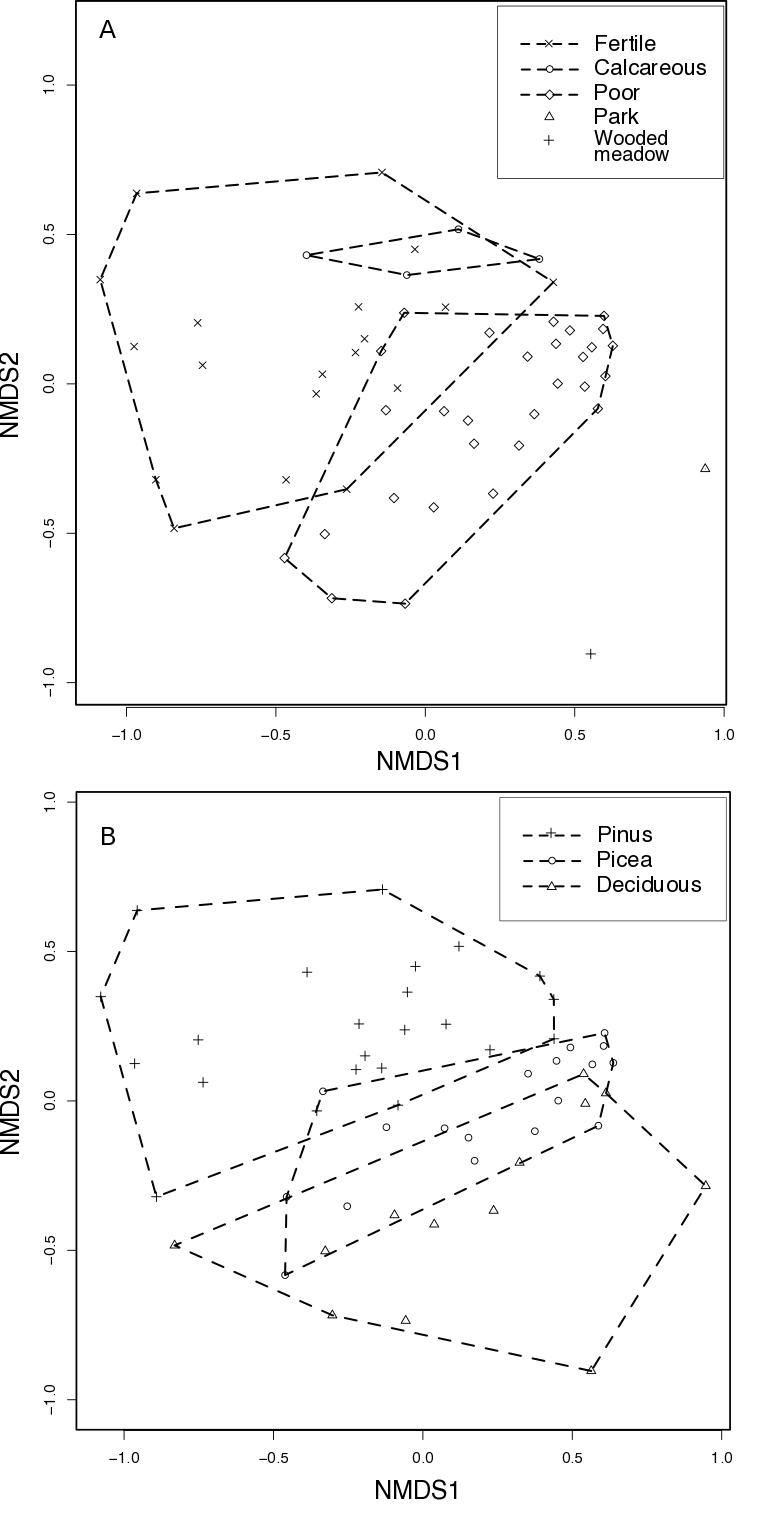

Supplement: Supplementary file 6 — Additional file 6. Non-metric multidimentional scaling (NMDS) ordination diagrams of polypore assemblages: (A) in forests on fertile and poor (excl. calcareous) soils and thin calcareous soils; (B) in woodlands with Picea and Pinus (including their mixedwood) or dominated by deciduous trees. [file 43008_2020_50_MOESM6_ESM.docx]
